# Using Class and Domain Information to Address Domain Shift in Federated Learning

**DOI:** 10.1109/TNNLS.2026.3658584

**Published:** 2026-09

**Authors:** Chien-Yu Chiou, Chun-Rong Huang, Lawrence L. Latour, Yang C. Fann, Pau-Choo Chung

**Affiliations:** Department of Electrical Engineering, National Cheng Kung University, Tainan 701, Taiwan; Department of Computer Science and the Institute of Multimedia Engineering, National Yang Ming Chiao Tung University, Hsinchu 300, Taiwan; Division of Intramural Research, National Institute of Neurological Disorders and Stroke, National Institutes of Health, Bethesda, MD 20814 USA; Division of Intramural Research, National Institute of Neurological Disorders and Stroke, National Institutes of Health, Bethesda, MD 20892 USA; Department of Electrical Engineering, National Cheng Kung University, Tainan 701, Taiwan; Department of Electrical Engineering, National Chung Cheng University, Chiayi 621, Taiwan

**Keywords:** Contrastive learning, domain shift, federated learning (FL), prototypes

## Abstract

In federated learning (FL), heterogeneous and client-specific data distributions cause a domain-shift problem, which leads to divergent local models and degraded global performance. To address this problem, this study proposes a class- and domain-aware FL framework that decouples and collaboratively learns the domain-invariant and domain-specific representations. During client training, a novel cross-gated feature separation (CGFS) module is employed to separate the domain features from the class features. A heterogeneous prototype contrastive learning (HPCL) module is then used to guide the learning of the class features and domain features with good discriminability within each feature space. Finally, during server aggregation, a gradient-reweighted hierarchical aggregation (GHA) strategy is applied to effectively aggregate information from all the clients and build a global model with good robustness to domain variation. The experimental results obtained on two FL datasets with domain shift show that the proposed method consistently outperforms state-of-the-art approaches.

## I. Introduction

Federated learning (FL) [1] is a promising strategy for preserving data privacy and represents a major paradigm shift in the artificial intelligence domain. The FL architecture typically consists of a server and multiple clients, and the training is conducted through multiple communication rounds that alternate between client training and server aggregation. In each communication round, the participating clients train local models using their own private data and then upload these models to the server. The server then aggregates these models to generate a global model. The global model is typically built through the weighted summation of the local models, in an approach equivalent to the summation of local gradients [1]. The global model is then dispatched to the clients for FL in the next round. In settings where the data distributions of the clients are independent and identically distributed (i.i.d.), the global model shows a comparable performance to that of normal deep learning models with access to all the data [1]. However, clients often have heterogeneous (non-i.i.d.) data distributions [2], which can degrade the performance of the model.

**Fig. 1.**
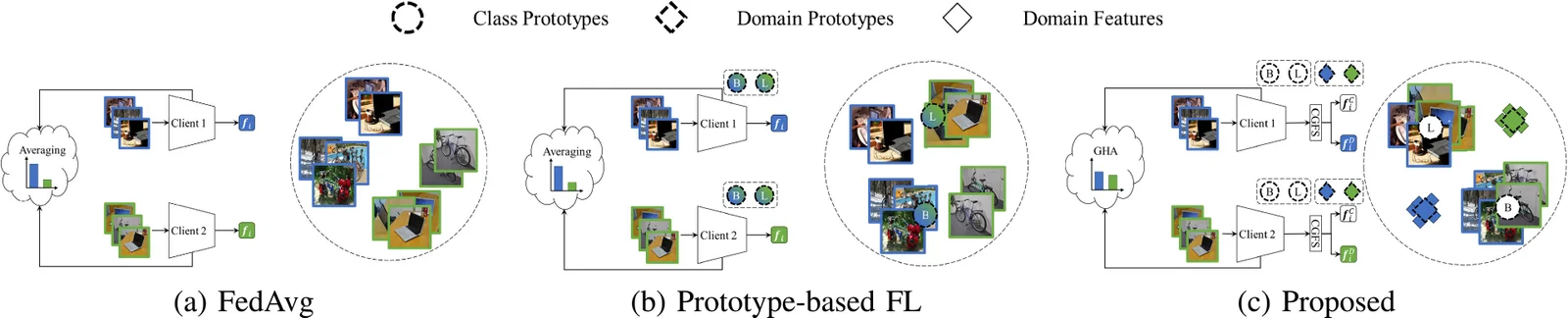
Comparison of conventional FL methods and proposed method. Clients 1 and 2 contain private data of the same classes (bicycle and laptop) of different domains. (a) Image features of the same class in different domains learned by FedAvg are separated, while the features of different classes in the same domain may be mixed together. (b) By aligning features with class prototypes, the features of the same class learned by prototype-based FL methods become closer, while the distribution is still affected by the domains. (c) Proposed method separates and learns class features and domain features individually by using the proposed CGFS module, class prototypes, and domain prototypes. Thus, the servers can aggregate class features of the same class from different domains by using the proposed GHA.

This article addresses a particularly challenging form of heterogeneous data distribution called the domain-shift problem [3], [4], [5], [6]. Fig. 1(a) illustrates a typical example of the considered problem. Client 1 captures images of bicycles and laptops using normal cameras in various uncontrolled environments. Consequently, the collected images have only moderate clarity and resolution and large variations in lighting, shading, and background. In contrast, Client 2 collects images using high-end digital single-lens reflex cameras in controlled environments. Thus, the collected images have high resolutions with clean backgrounds and minimal variations in lighting and shading. Although the class is the same for both clients, the visual appearance of the objects in the images is strongly shaped by the data collection context. Therefore, the local datasets encode not only the class information but also the domain information. When local models are trained independently on such divergent domains, the aggregated global model struggles to align these representations. This leads to significant performance degradation, the core of the domain-shift problem in FL.

Moreover, under domain shift, the learned features inevitably entangle class- and domain-relevant information. For example, as shown in Fig. 1(a), the learned features belonging to the same class but coming from different domains are separated, while the features belonging to different classes but in different domains overlap. In this case, the features represent the domains rather than the classes, making it difficult for the model to discriminate among the different objects. In addition, each client fits its local model using its own private data during client training. As a result, the model gradients from different clients diverge significantly and convey highly domain-dependent learning signals. If the server then aggregates these gradients weighted purely by the volume of data at each client, the global model becomes biased toward the clients with dominant data volumes and may underfit those with fewer data [4], [7]. Therefore, mitigating domain shift in both feature representation learning and model aggregation is essential for improving the performance and fairness of FL.

However, in the FL setting, clients do not have access to the data of other domains. Thus, conventional domain adaptation methods [8], domain generalization methods [9], and transformation-invariant feature methods [10] that rely on centralized access to multidomain data to learn domain-invariant features cannot be directly applied.

Many prototype-based FL methods have been proposed to alleviate this problem [3], [11], [12], [13]. As shown in Fig. 1(b), these methods aim to improve the model performance by aligning features of the same class using class prototypes, where these prototypes are usually calculated by averaging the features of each client. However, existing prototype-based methods focus only on semantic class information while ignoring domain characteristics. Thus, the learned features of different clients for the same class may still be affected by the domain information. In addition, only the features are aligned with the class prototypes, while the local model parameters remain divergent across clients. As a result, the models from different clients still contain diverse domain information. Consequently, when aggregating the global model at the server, the diverse directions of the local models may still cause the model to be biased toward the majority data. Thus, despite prototype-based alignment, domain shift remains unresolved in these methods.

To solve these problems, the present study proposes a class- and domain-information-aware FL framework that explicitly separates and learns class-discriminative and domain-specific representations during client training, as shown in Fig. 1(c). The learned information is then jointly utilized to help generate a more reliable global model during server aggregation. By properly disentangling and integrating the class and domain cues, features belonging to the same class across different domains can be aligned more accurately, thereby substantially reducing the impact of the domain-shift problem.

**Fig. 2.**
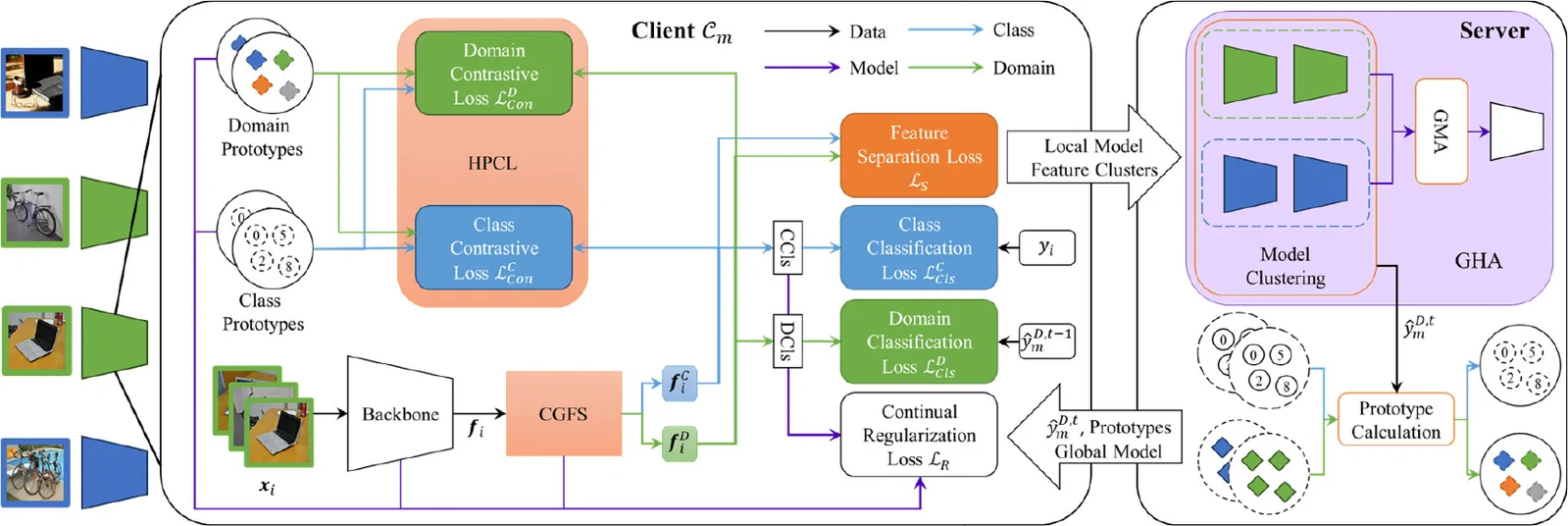
Overview of the proposed method. In each client, the backbone extracts the features $f_{i}$ of the image $x_{i}$. The proposed CGFS module learns to separate the image features $f_{i}$ into class features $f_{i}^{C}$ and domain features $f_{i}^{D}$ using the feature separation loss ${ℒ}_{S}$. Then, the proposed HPCL module guides feature learning by using the class contrastive loss ${ℒ}_{\text{Con}}^{C}$ and domain contrastive loss ${ℒ}_{\text{Con}}^{D}$ with the class prototypes and domain prototypes. The class classifiers (CCls) and domain classifiers (DCls) learn to identify the class and domain by using the class classification loss ${ℒ}_{Cls}^{C}$ and domain classification loss ${ℒ}_{Cls}^{D}$ based on the class label $y_{i}$ and the server-assigned domain label ${\overset{ˆ}{y}}_{m}^{D,t-1}$, respectively. The continual regularization loss ${ℒ}_{R}$ constrains the model update direction. After client training, the local models and feature clusters of each client are uploaded to the server to build the global model. The server first clusters the client models and assigns domain labels ${\overset{ˆ}{y}}_{m}^{D,t}$ based on the parameters of the local models. Then, the global model is built by using the proposed GMA method and local model clusters. After model aggregation, the global class prototypes and global domain prototypes are calculated based on ${\overset{ˆ}{y}}_{m}^{D,t}$ and the feature clusters from the clients.

To learn the domain information and class information from each client, a novel client-side learning network is proposed, as shown in Fig. 2. The framework consists of a cross-gated feature separation (CGFS) module and a heterogeneous prototype contrastive learning (HPCL) module. The CGFS module disentangles the features of the input images into class features and domain features. In each client, the class features encode the semantic cues needed for object recognition, while the domain features capture the appearance variations caused by the client-specific imaging conditions (e.g., resolution, background, and illumination). During server aggregation, the class features and domain features of the different clients are aggregated to generate separate class prototypes and domain prototypes, respectively. These prototypes are then transmitted back to each client to help the HPCL module learn consistent class features and domain features in subsequent rounds of the FL process.

To drive client training, a feature separation loss is first applied to distinguish the class features from the domain features. To enhance the discriminability of the separated features, a class contrastive loss and class classification loss are then used to reinforce the semantic clustering of the class features. Similarly, a domain contrastive loss and a domain classification loss are utilized to learn the representative domain features. Finally, a continual regularization loss is applied to stabilize the local model updates and mitigate overfitting to private client data. After client training, the class features and domain features in each client are clustered to represent the feature distribution of the client. The local models and clustered features are then uploaded to the server, as shown in Fig. 2.

To prevent the global model from being biased toward the clients with dominant data volumes, this study further proposes a gradient-reweighted hierarchical aggregation (GHA) method on the server side. First, the local client models are clustered in parameter space to identify groups with similar domain characteristics. The server then aggregates models within the same domain cluster to produce domain-specific models, which are then combined to form a global model. To effectively aggregate the local models, a gradient-aware model aggregation (GMA) strategy is employed to adjust the summation weights based on both the gradients of the local models and their relative data volumes. After model aggregation, the global class prototypes and global domain prototypes are calculated using the clustered feature distributions of all the clients. Finally, the global model and global prototypes are dispatched to the clients for FL training in the next round. By jointly optimizing the client-side representation learning and server-side aggregation processes, the proposed framework more effectively integrates the heterogeneous domain information, thus improving the overall performance of the FL model.

The contributions of this study can be summarized as follows.

1. First, to address the domain-shift problem in FL, a novel CGFS module and HPCL module are proposed to separate and learn the domain-specific features and class-discriminative features independently.
2. Second, a GHA method is introduced on the server side to more effectively integrate information from clients with different domains.
3. Third, extensive experiments on two FL datasets with domain-shift settings confirm the superiority of the proposed method compared with state-of-the-art methods.

The remainder of this article is organized as follows. Section II discusses related research on FL with heterogeneous client data. Section III introduces the proposed framework in detail. Section IV presents and discusses the experimental results. Finally, Section V provides some brief conclusions.

## II. Related Work

### A. FL for Heterogeneous Data

To address the problem of heterogeneous data distribution in FL, several methods have proposed constraining the update directions of the client models to enforce greater consistency among them [7], [14], [15], [16], [17], [18], [19], [20], [21]. However, these techniques generally rely on the previously aggregated global model to regularize the client updates. Overly constraining the local models may limit their ability to learn new data, while insufficient constraints may fail to prevent divergence across clients, leading to persistent heterogeneity in the learned models.

Some methods attempt to improve the integration of the information received from different clients during server aggregation by adjusting the summation weights or clipping part of the gradients [4], [22], [23], [24]. For example, the summation weights of the local models may be adjusted based on criteria such as their performance [23], loss [24], or temporal gradient consistency [4]. Li et al. [25] found that global models with smaller norms show better generalization ability and accordingly introduced a learnable global shrinking factor to improve the generalization ability of the global model. Shi et al. [26] proposed a layer-wise adjustment of the global shrinking factor to further improve the performance. To improve model consensus, FedAWA [27] adaptively increases the aggregation weights of the local models closer to the current global model in the parameter space.

Farhadkhani et al. [28] applied static clipping to the local model gradients to improve the robustness of the model toward false gradients sent by adversarial clients. In a more recent study, Allouah et al. [29] proposed an adaptive clipping method to further improve the model robustness. However, in the absence of additional constraints during client training, the updated gradients of the different local models are still diverse due to the domain-shift problem. The diverse gradients conflict with each other and lead to less effective aggregation at the server when constructing the global model.

Another line of research leverages knowledge of the data distributions of the other clients during local training. To preserve data privacy, this information is typically transferred through surrogate representations, such as heuristic mathematical models specific to the particular problem [30], feature decomposition matrices [31], adaptive augmentation policies [32], learned offset models [33], or generative models [34], [35]. Other approaches generate augmentation samples to simulate the data distributions of the other clients. For example, Zhou et al. [5] generated local training samples based on the statistics of the intermediate layer feature distributions of the other clients. Similarly, FedRDN [36] randomly mixes the mean and standard deviation between clients to diversify the training space. By transferring data distributions, these methods help clients exploit the data distributions of the other clients to enhance their training performance. However, heuristic models may not fully represent the nonlocal data distributions. Furthermore, the need for offset models and generative models increases the computational cost and remains susceptible to domain shift, particularly when the client distributions differ substantially.

Some recent studies have built multiple global models to specifically fit the data of each domain or client rather than learning a single universal model. For instance, in [37], [38], and [39], the server clusters the clients into multiple domains and builds a cluster model for each cluster based on the local models within the same cluster. However, this approach requires the server to maintain multiple models rather than just one. Furthermore, the cluster models learn little information from the clients in different clusters, thereby limiting the ability of the model to generalize across domains. In a different approach, studies [40], [41], [42], [43], [44], [45] build additional personal adaptive models in clients to personalize the shared global model to the private data of each client. In a recent study [6], domain-specific skew erasers were proposed to suppress domain-specific features and enhance feature consensus. Although personalization-based methods improve adaptation to private client data, they require the clients to train and maintain additional personal models, and the global model may still be impaired by the domain-shift problem.

The personalized FL process can be formulated as a meta-learning procedure that aims to learn a model capable of rapid adaptation to different tasks [46]. In such methods, the server builds a global metamodel using the information provided by the participating clients, and each client then personalizes this model based on its own private data. AFM3D [47] applies a first-order approximation to the client model gradients to reduce the computational cost of meta learning. Similarly, Wang et al. [48] limit meta learning to only the participating clients and predict the updates of the nonparticipating clients using an attention-based model. You et al. [49] proposed a framework for personalizing Internet of Things (IoT) models using federated meta learning. Unlike centralized meta-learning, where full access to multitask data and gradients is available, federated meta-learning approaches require client updates to be performed locally and model aggregation to occur with limited information sharing. This leads to higher system complexity and substantial challenges in efficiently training models across distributed devices. This, in turn, may adversely affect the convergence and overall performance of the model [46].

Since FL clients have no access to the data of other clients, catastrophic forgetting may occur during local training, causing the client models to forget knowledge learned in previous rounds [19]. To solve this problem, federated incremental learning methods have been proposed that incrementally learn new unseen tasks while retaining the knowledge acquired from previous tasks. For example, Li et al. [50] trained a set of personalized local models at each client, with each model corresponding to at least one domain task. The final predictions were then aggregated based on a knowledge-matching score across tasks. Zhang et al. [51] assigned unique orthogonal frequency components to different clients and learned personalized local models independently to avoid interference that might induce catastrophic forgetting. ReFed [52] built a personalized informative model at each client to estimate the importance scores of the training samples and then selected replay samples based on both importance and global class distribution statistics.

Since clients cannot share cached data, cache-based federated incremental learning methods are effective only when the clients encounter new unseen tasks. Furthermore, personalization-based methods require the clients to train and maintain additional personal models, which increases the computation and storage costs at the client.

### B. Prototype-Based FL

More recently, prototype-based methods have been proposed that employ prototypes as anchors for class-discriminative features during learning. In particular, clients align their learned features to server-provided prototypes throughout training. Some methods predefine the prototypes using handcrafted formulations or pretrained models [53], [54]. For example, Shi et al. [54] adopted a pretrained CLIP model [55] to define class prototypes. However, predefined prototypes may not accurately represent the real feature distributions. Moreover, they are not guaranteed to fit the evolving data at the client during training. Consequently, other methods compute the prototypes dynamically based on the features of each class provided by the participating clients [3], [11], [12], [13], [43], [56], [57], [58], [59], [60], [61], [62]. FedGMKD [58] builds representative prototypes and soft predictions of each class using a Gaussian mixture model (GMM) and then adjusts the model aggregation weights based on the quality and quantity of the prototypes and soft predictions. An et al. [62] further improved feature alignment by leveraging correlations among semantically related classes.

To further enhance the discriminability among different classes, several prototype-based contrastive learning methods have been proposed [3], [56], [63]. For example, Huang et al. [3] increased the interclass discriminability by performing contrastive learning using cluster prototypes and enhanced the feature consistency within the same class using unbiased prototypes. Wang et al. [12] proposed an α-sparsity prototype loss to accommodate the distribution sparsity of interclass features. To further enhance the relationship between the features and prototypes, pFedCSPC [43] regularized the relative angles and distances between the features and prototypes of different classes. FedSA [60] employs predefined class anchors and margin-enhanced contrastive learning to enforce class boundaries at the client side and updates these anchors at the server based on the exponential moving average of the client feature statistics. FedMPS [64] proposes to build multilevel prototypes to capture high-level semantic features and detailed low-level features.

Rather than attempting to guide the client feature training process, some methods focus on generating well-separated prototypes on the server side. FedTGP [13] trains a global prototype generator on the server based on the client features using a contrastive learning approach with an adaptive interclass margin. FedLSA [59] enhances server-side prototype separability by applying a similarity-based separation loss, supported by a global semantic-aware classifier.

Feature-based prototypes provide information about the underlying feature distribution and therefore improve feature learning. However, they typically consider only the class information. Consequently, the local models and features learned by different clients may still retain domain-specific variations and remain diverse, leading to the domain-shift problem. Moreover, the global model and prototypes generated through simple averaging of the diverse local models and features may be biased toward clients with dominant data volumes.

To address the domain-shift problem, the method proposed in this study learns the class information and the domain information individually. In particular, the CGFS module first disentangles the domain features from the class features at the client side. The HPCL module then enforces consistency among the class features belonging to the same semantic category, thereby reducing divergence among the client models during local training. Finally, the heterogeneous local models are effectively aggregated at the server side using the GHA strategy based on the domain characteristics and gradient behavior. Overall, through joint optimization on both the client side and the server side, the proposed framework mitigates the domain-shift problem and thus improves performance on FL tasks involving heterogeneous data distributions.

## III. Method

### A. Problem Definition

Let 𝒮 represent the server in FL and ${\left\{{𝒞}_{m}\right\}}_{m=1}^{M}$ represent a set of M participating clients. Each client has its own private data ${𝒟}_{m}={\left\{\left(x_{i},y_{i}\right)\right\}}_{i=1}^{N_{m}}$, where $x_{i}$ and $y_{i}$ are the *i*th image and its class label, and $N_{m}$ is the total number of images. The aim of the FL process is to train a global model F(⋅) with parameters θ that fit the data of all the clients through T successive training rounds. Each round involves a client training stage and a server aggregation stage. During the client training stage of the t th round, the m th client trains a local model $F_{m}^{t}(\cdot )$ with parameters $\theta_{m}^{t}$ to minimize the training loss ${ℒ}_{m}^{t}$ in the private data ${𝒟}_{m}$ as follows:

(1) $$\theta_{m}^{t*}=arg\;\underset{\theta_{m}^{t}}{min}\sum_{x_{i},y_{i}\in {𝒟}_{m}}{ℒ}_{m}^{t}\left(F_{m}^{t}\left(x_{i};\theta_{m}^{t}\right),y_{i}\right).$$

After client training, the local models are sent to the server to build a global model with parameters $\theta^{t}$ via the following weighted summation:

(2) $$\theta^{t}=\sum_{m=1}^{M}w_{m}\theta_{m}^{t*},\;\text{s.t.}\;\sum_{m=1}^{M}w_{m}=1$$

where $w_{m}$ is the summation weight of the m th local model.

Since the data collected from different clients usually have different characteristics, the data distributions $P_{m}(x|y)$ and $P_{n}(x|y)$ of two different clients may be different for the same class y, as formulated in the following:

(3) $$P_{m}\left(x|y\right)\neq P_{n}\left(x|y\right),\;\text{s.t.}\;P_{m}(y)=P_{n}(y).$$

These different distributions imply that the data contain not only class information but also domain information. Local models trained on data with different distributions will inevitably diverge, leading to the well-known domain-shift problem [3], [4], [5], [6]. This divergence poses a significant challenge, as the server must aggregate these heterogeneous local models into a single global model capable of representing the data from all the clients, regardless of the domain differences between them.

### B. Overview

To solve the domain-shift problem, the FL framework proposed in the present study learns the class information and the domain information separately during the client training stage and then hierarchically aggregates the local models of the clients from different domains using a gradient-aware aggregation strategy, as shown in Fig. 2. During training at client ${𝒞}_{m}$, the proposed CGFS module learns to disentangle the class features and domain features of the local images. The proposed HPCL module then guides feature learning through the use of dual-class prototypes ${𝒫}_{m}^{t}=\left\{{𝒫}_{m}^{1,t},{𝒫}_{m}^{2,t}\right\}$ and dual-domain prototypes ${𝒬}_{m}^{t}=\left\{{𝒬}_{m}^{1,t},{𝒬}_{m}^{2,t}\right\}$ provided by the server. The first group prototypes, ${𝒫}_{m}^{1,t}$ and ${𝒬}_{m}^{1,t}$, are adaptively learned in round t based on private data during client training, while the second group prototypes, ${𝒫}_{m}^{2,t}$ and ${𝒬}_{m}^{2,t}$, are provided by the server in round t to serve as fixed anchors. Based on ${𝒫}_{m}^{t}$ and ${𝒬}_{m}^{t}$, the HPCL module learns the representative class features and domain features of the private data during client training, and these learned features are then clustered to represent the class and domain feature distributions of the client. Finally, the local models and clustered features are uploaded to the server for aggregation.

During server aggregation, the proposed GHA strategy is employed to prevent the global model from being biased toward the clients with dominant data volumes. The server first identifies the domains ${\overset{ˆ}{y}}_{m}^{D,t}$ of the participating clients by clustering the model parameters. It then aggregates the local models within the same domain to construct a model for each domain. Finally, the server builds a global model by aggregating the individual domain models. To ensure effective integration of the heterogeneous local models, the summation weights in the hierarchical aggregation process are adaptively adjusted using the proposed GMA strategy. After model aggregation, the server calculates the global class prototypes ${𝒫}^{t}$ and global domain prototypes ${𝒬}^{t}$ based on the feature cluster information provided by the clients. Finally, the global model and global prototypes are dispatched to the clients for FL training in the next round.

Based on the separated class and domain information, the client training objective is defined as follows:

(4) $$\begin{array}{l} \theta_{m}^{t*},{𝒫}_{m}^{1,t*},{𝒬}_{m}^{1,t*}\\ \;=\underset{\theta_{m}^{t},{𝒫}_{m}^{1,t},{𝒬}_{m}^{1,t}}{argmin}\sum_{x_{i},y_{i}\in {𝒟}_{m}}{ℒ}_{m}^{t}\left(F_{m}^{t}\left(x_{i};\theta_{m}^{t},{𝒫}_{m}^{t},{𝒬}_{m}^{t}\right),y_{i},{\overset{ˆ}{y}}_{m}^{D,t}\right). \end{array}$$

During server aggregation, the GHA strategy is employed to cluster the client model parameters to infer the domain of each client ${\overset{ˆ}{y}}_{m}^{D,t}$ and domain-specific models are then constructed using the parameters $\theta^{d,t}$ of each domain d as follows:

(5) $$\theta^{d,t}=\sum_{\left\{m|{\overset{ˆ}{y}}_{m}^{D,t}=d\right\}}w_{m}^{d}\theta_{m}^{t*},\;\text{s.t.}\;\sum_{\left\{m|{\overset{ˆ}{y}}_{m}^{D,t}=d\right\}}w_{m}^{d}=1$$

where $w_{m}^{d}$ are the summation weights calculated based on the amount of data and model gradients of the clients belonging to domain d. The global model with parameters $\theta^{t}$ is then obtained as follows:

(6) $$\theta^{t}=\sum_{d}w^{d}\theta^{d,t},\;\text{s.t.}\;\sum_{d}w^{d}=1$$

where $w^{d}$ is the summation weight calculated based on the amount of data and model gradients of the corresponding domain model.

### C. Client Training

The proposed client training stage and the associated network structure are shown in Fig. 2. It is assumed that the server assigns a client ${𝒞}_{m}$ with private training dataset ${𝒟}_{m}$ to domain ${\overset{ˆ}{y}}_{m}^{D,t-1}$. For each training image $x_{i}$ of class $y_{i}$, the backbone extracts a set of deep features $f_{i}$ to represent the image. In accordance with the domain-shift problem, the data distributions of the images belonging to the same class but different domains are different because the images contain not only class information but also domain information. The extracted features $f_{i}$ also contain both class information and domain information. Therefore, the present study uses the CGFS module to separate $f_{i}$ into class features $f_{i}^{C}$ and domain features $f_{i}^{D}$. Based on these features, the CCls and DCls learn to predict the class and domain of the image.

The features of the images belonging to the same class should exhibit high consistency, whereas those from different classes should be clearly distinguishable. To improve the learned features, the HPCL module is employed to guide feature learning by leveraging heterogeneous dual-class prototypes and dual-domain prototypes. Accordingly, several loss functions are introduced to drive the client-side optimization. First, a feature separation loss ${ℒ}_{S}$ guides the CGFS module to disentangle the class features and domain features by enforcing orthogonality between $f_{i}^{C}$ and $f_{i}^{D}$. A class contrastive loss ${ℒ}_{\text{Con}}^{C}$ is then applied together with a domain contrastive loss ${ℒ}_{\text{Con}}^{D}$ to learn representative class features and domain features with the assistance of the HPCL module, class prototypes, and domain prototypes. To distinguish images of different classes, a class classification loss ${ℒ}_{\text{Cls}}^{C}$ is applied. Similarly, the model learns to identify the domain features of different domains using a domain classification loss ${ℒ}_{\text{Cls}}^{D}$. Finally, a continual regularization loss ${ℒ}_{R}$ is employed to constrain the updating of the local models by penalizing deviations of the model from the global model constructed in the previous aggregation round.

After client training, the class features and domain features at each client are clustered to represent the feature distribution of the client. Finally, the local model, clustered class features, and clustered domain features of each client are sent to the server 𝒮. The details of the CGFS module, HPCL module, training loss functions, and feature clustering are presented in the following subsections.

#### 1) CGFS Module:

**Fig. 3.**
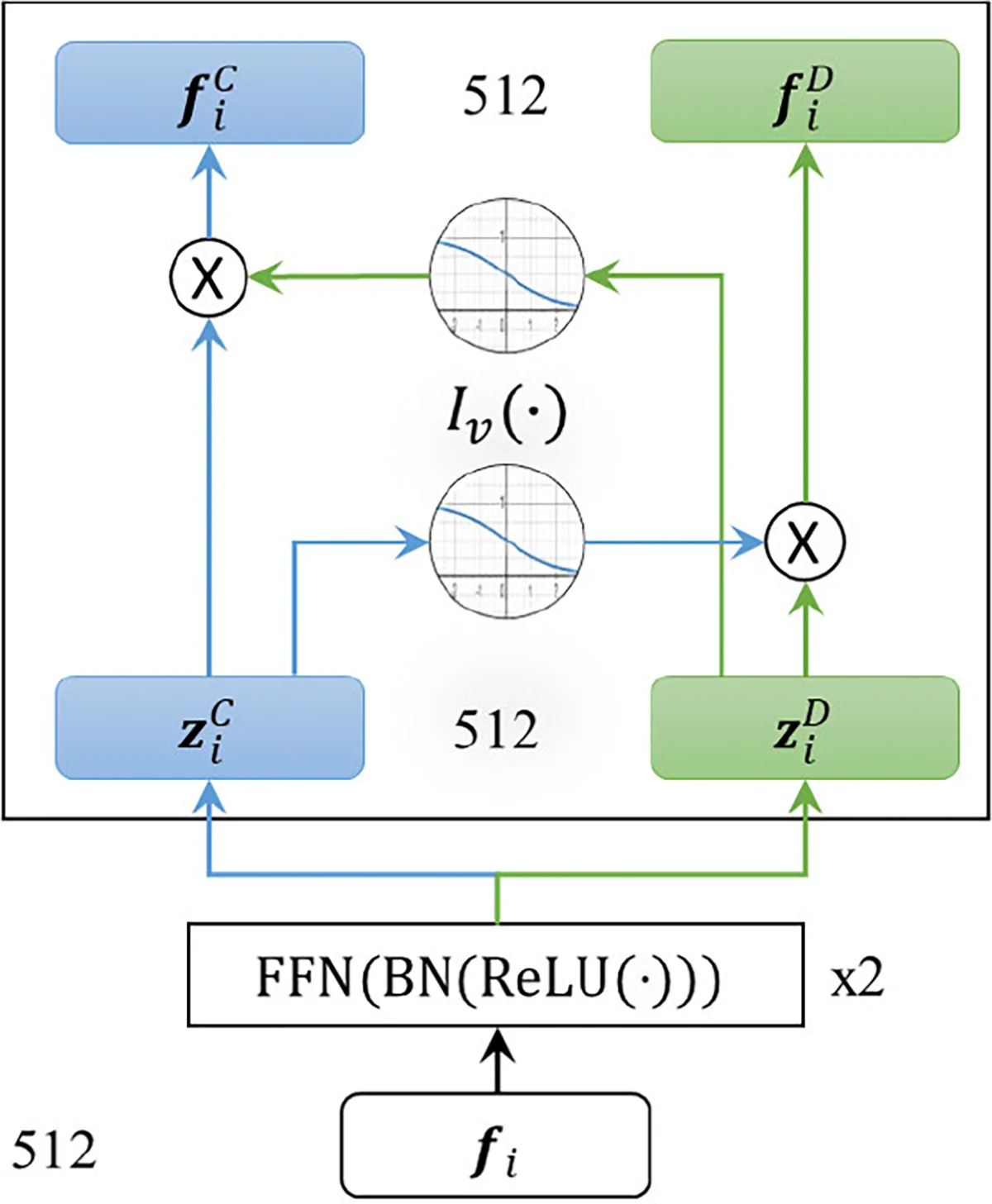
Proposed CGFS module. The image features $f_{i}$ are first projected into class features $z_{i}^{C}$ and domain features $z_{i}^{D}$. Then, the proposed inverse gating function $I_{v}(\cdot )$ enforces the feature separation by suppressing the responses of the class features based on the domain features and vice versa for the domain features. The final class features $f_{i}^{C}$ and domain features $f_{i}^{D}$ are generated by using $z_{i}^{C}$, $z_{i}^{D}$, and $I_{v}(\cdot )$.

As formulated in (3), images belonging to the same class but different domains exhibit distinct data distributions. The domain characteristics thus affect the features representing the images and cause feature diversity within the same semantic class. To address this issue, the CGFS module is employed to separate the domain features from the class features and learn the two types of features independently. Fig. 3 shows the architecture of the CGFS module. Two feed-forward layers first project the image features $f_{i}$ into class features $z_{i}^{C}$ and domain features $z_{i}^{D}$. To ensure effective feature disentanglement, the feature dimensions are explicitly separated from those associated with domain information. Specifically, if a dimension has high responses in the class features, the same dimension should have low or zero responses in the domain features and vice versa. To enforce this separation and suppress conflicting responses, the following inverse gating function $I_{v}(\cdot )$ is introduced:

(7) $$I_{v}(z)=1-\sigma (z)$$

where $\sigma (z)=1/\left(1+e^{-z}\right)$ is the sigmoid function. $I_{v}(\cdot )$ is then applied to the two feature components such that each component suppresses the dimensions associated with the other, formulated as follows:

(8) $$f_{i}^{C}=z_{i}^{C}\cdot I_{v}\left(z_{i}^{D}\right)$$

and

(9) $$f_{i}^{D}=z_{i}^{D}\cdot I_{v}\left(z_{i}^{C}\right).$$

To encourage the separation of the class features and domain features, the following orthogonal-based feature separation loss ${ℒ}_{S}$ is proposed:

(10) $${ℒ}_{S}=\sum_{x_{i}\in {𝒟}_{m}}\left|sim\left(f_{i}^{C},f_{i}^{D}\right)\right|.$$

In this way, the CGFS module not only enforces the separation of the class and domain features but also encourages the model to improve the learned features.

#### 2) HPCL Module:

Through the CGFS module, the client model learns the class features and domain features individually. To further ensure consistency among the features of images from the same class, the HPCL module leverages dual-class prototypes ${𝒫}_{m}^{t}=\left\{{𝒫}_{m}^{1,t},{𝒫}_{m}^{2,t}\right\}$ and dual-domain prototypes ${𝒬}_{m}^{t}=\left\{{𝒬}_{m}^{1,t},{𝒬}_{m}^{2,t}\right\}$. The prototypes are provided by the server and represent the distributions of the class features and domain features, respectively. The HPCL module learns representative class features and domain features during client training, utilizing a contrastive learning approach [65], [66], [67]. In contrastive learning of the class features, the class features of each image form a positive pair set with the corresponding class prototypes and a negative pair set with the other prototypes. The contrastive loss then aligns the class features toward the prototypes of the positive pair set while simultaneously pushing them away from those in the negative pair set. The domain features are learned in a similar manner.

Let ${𝒫}_{m}^{j,t}={\left\{\left(p_{m,k}^{j,t},u_{m,k}^{j,t}\right)\right\}}_{k=1}^{\eta_{m}^{j,t}}$ and ${𝒬}_{m}^{j,t}={\left\{\left(q_{m,k}^{j,t},v_{m,k}^{j,t}\right)\right\}}_{k=1}^{\xi_{m}^{j,t}}$ for j∈{1,2}, where $p_{m,k}^{j,t}$, $u_{m,k}^{j,t}$, and $\eta_{m}^{j,t}$ are the *k*th class prototype, the *k*th prototype label, and the number of class prototypes in ${𝒫}_{m}^{j,t}$, and $q_{m,k}^{j,t}$, $v_{m,k}^{j,t}$, and $\xi_{m}^{j,t}$ are the *k*th domain prototype, the *k*th prototype label, and the number of domain prototypes in ${𝒬}_{m}^{j,t}$, respectively. Given an image $x_{i}$ belonging to class $y_{i}$ at client ${𝒞}_{m}$, the client model backbone and CGFS module extract the class features $f_{i}^{C}$ and domain features $f_{i}^{D}$. The class features should be similar to the prototypes of the same class while being dissimilar to those of different classes. In addition, to enhance feature separation and mitigate the domain-shift problem, the class features should also be dissimilar to the domain prototypes. Thus, class feature learning is guided by the following class contrastive loss ${ℒ}_{\text{Con}}^{C}$ as follows:

(11) $${ℒ}_{Con}^{C}={ℒ}_{Con}^{C,F}+{ℒ}_{Con}^{C,P}$$

where ${ℒ}_{Con}^{C,F}$ updates the class features of the client model, while ${ℒ}_{\text{Con}}^{C,P}$ updates the class prototypes of the first group. To compute ${ℒ}_{\text{Con}}^{C,F}$, a positive pair set $\Psi_{i}^{C,F}$ and a negative pair set $\Omega_{i}^{C,F}$ are first formed for $f_{i}^{C}$. $\Psi_{i}^{C,F}$ contains positive pairs from the class features $f_{i}^{C}$ and class prototypes of the same class $y_{i}$ and is defined as follows:

(12) $$\Psi_{i}^{C,F}=\left\{\left(f_{i}^{C},p_{m,k}^{j,t}\right)|u_{m,k}^{j,t}=y_{i}\;\forall \;j\in \{1,\;2\}\right\}.$$

Meanwhile, $\Omega_{i}^{C,F}$ contains negative pairs that should be dissimilar. These pairs are constructed using the class feature set $f_{i}^{C}$, class prototypes whose labels are not $y_{i}$ and the domain prototypes. $\Omega_{i}^{C,F}$ is defined as follows:

(13) $$\begin{array}{r} \Omega_{i}^{C,F}=\left\{\left(f_{i}^{C},p_{m,k}^{j,t}\right)|u_{m,k}^{j,t}\neq y_{i}\forall \;j\in \{1,\;2\}\right\}\\ \cup \left\{\left(f_{i}^{C},q_{m,r}^{j,t}\right)|\forall \;j\in \{1,\;2\}\right\}. \end{array}$$

Let $sim\left(v_{1},v_{2}\right)$ be the cosine similarity between two vectors $v_{1}$ and $v_{2}$ and let $S\left(v_{1},v_{2}\right)=exp\left(\left(sim\left(v_{1},v_{2}\right)\right)/\tau \right)$, where τ=0.5 is the temperature parameter. The class contrastive loss ${ℒ}_{\text{Con}}^{C,F}$ is then computed based on $\Psi_{i}^{C,F}$ and $\Omega_{i}^{C,F}$ as follows:

(14) $${ℒ}_{\text{Con}}^{C,F}=-\sum_{x_{i}\in {𝒟}_{m}}log\frac{\sum_{\left(\mu_{1},\mu_{2}\right)\in \Psi_{i}^{C,F}}\;S\;\left(\mu_{1},\mu_{2}\right)}{\sum_{\left(v_{1},\;v_{2}\right)\in \Psi_{i}^{C,F}\cup \Omega_{i}^{C,F}}\;S\;\left(v_{1},\;v_{2}\right)}.$$

To compute ${ℒ}_{\text{Con}}^{C,P}$, a positive pair set $\Psi_{i}^{C,P}$ and negative pair set $\Omega_{i}^{C,P}$ are first formed. The positive pair set $\Psi_{i}^{C,P}$ consists of positive pairs formed between the first-group class prototype $p_{m,k}^{1,t}$ and both the class features $f_{i}^{C}$ and the remaining class prototypes of the first and second groups that share the same class label $y_{i}$. It is defined as follows:

(15) $$\begin{array}{ll} \Psi_{i}^{C,P} & =\left\{\left(p_{m,k}^{1,t},f_{i}^{C}\right)|u_{m,k}^{1,t}=y_{i}\right\}\\ \; & \cup \left\{\left(p_{m,k}^{1,t},p_{m,r}^{1,t}\right)|u_{m,k}^{1,t}=y_{i},u_{m,r}^{1,t}=y_{i},k\neq r\right\}\\ \; & \cup \left\{\left(p_{m,k}^{1,t},p_{m,s}^{2,t}\right)|u_{m,k}^{1,t}=y_{i},u_{m,s}^{2,t}=y_{i}\right\}. \end{array}$$

The negative pair set $\Omega_{i}^{C,P}$ consists of negative pairs formed between the first-group class prototype $p_{m,k}^{1,t}$ and the remaining class prototypes of the first or the second group whose labels are not $y_{i}$, together with the domain prototypes. It is defined as follows:

(16) $$\begin{array}{ll} \Omega_{i}^{C,P} & =\left\{\left(p_{m,k}^{1,t},p_{m,r}^{j,t}\right)|u_{m,k}^{1,t}=y_{i},u_{m,r}^{j,t}\neq y_{i}\forall \;j\in \{1,\;2\}\right\}\\ \; & \cup \left\{\left(p_{m,k}^{1,t},q_{m,s}^{j,t}\right)|\forall \;j\in \{1,\;2\}\right\}. \end{array}$$

Then, ${ℒ}_{\text{Con}}^{C,P}$ is computed based on $\Psi_{i}^{C,P}$ and $\Omega_{i}^{C,P}$ as follows:

(17) $${ℒ}_{\text{Con}}^{C,P}=-\sum_{x_{i}\in {𝒟}_{m}}log\frac{\sum_{\left(\mu_{1},\mu_{2}\right)\in \Psi_{i}^{C,P}}\;S\;\left(\mu_{1},\;\mu_{2}\right)}{\sum_{\left(v_{1},v_{2}\right)\in \Psi_{i}^{C,P}\cup \Omega_{i}^{C,P}}\;S\;\left(v_{1},\;v_{2}\right)}.$$

The class contrastive loss guides the client model to reduce the distance between class features belonging to the same class while increasing the distance between class features of different classes and all the domain-related features. Thus, the client model learns class features that are both representative of their semantic categories and highly discriminative from other classes, leading to an improved classification performance.

Similarly, to learn the domain features, the domain contrastive loss ${ℒ}_{\text{Con}}^{D}$ is calculated as follows:

(18) $${ℒ}_{\text{Con}}^{D}={ℒ}_{\text{Con}}^{D,F}+{ℒ}_{\text{Con}}^{D,P}$$

where ${ℒ}_{\text{Con}}^{D,F}$ updates the domain features of the client model and ${ℒ}_{\text{Con}}^{D,P}$ updates the domain prototypes of the first group. To compute ${ℒ}_{\text{Con}}^{D,F}$, a positive pair set $\Psi_{i}^{D,F}$ and negative pair set $\Omega_{i}^{D,F}$ for $f_{i}^{D}$ are formed. $\Psi_{i}^{D,F}$ consists of positive pairs of the domain features and domain prototypes of domain ${\overset{ˆ}{y}}_{m}^{D,t-1}$ as follows:

(19) $$\Psi_{i}^{D,F}=\left\{\left(f_{i}^{D},q_{m,k}^{j,t}\right)|v_{m,k}^{j,t}={\overset{ˆ}{y}}_{m}^{D,t-1}\;\forall \;j\in \{1,\;2\}\right\}.$$

$\Omega_{i}^{D,F}$ consists of negative pairs formed by the domain features $f_{i}^{D}$, domain prototypes with labels different to ${\overset{ˆ}{y}}_{m}^{D,t-1}$, and class prototypes as follows:

(20) $$\Omega_{i}^{D,F}=\left\{\left(f_{i}^{D},q_{m,k}^{j,t}\right)|v_{m,k}^{j,t}\neq {\overset{ˆ}{y}}_{m}^{D,t-1}\;\forall \;j\in \{1,\;2\}\right\}\;\;\cup \left\{\left(f_{i}^{D},p_{m,r}^{j,t}\right)|\forall \;j\in \{1,\;2\}\right\}$$

based on $\Psi_{i}^{D,F}$ and $\Omega_{i}^{D,F}$, the domain contrastive loss ${ℒ}_{\text{Con}}^{D,F}$ is computed as follows:

(21) $${ℒ}_{\text{Con}}^{D,F}=-\sum_{x_{i}\in {𝒟}_{m}}log\frac{\sum_{\left(\mu_{1},\mu_{2}\right)\in \Psi_{i}^{D,F}}\;S\;\left(\mu_{1},\;\mu_{2}\right)}{\sum_{\left(v_{1},v_{2}\right)\in \Psi_{i}^{D,F}\cup \Omega_{i}^{D,F}}\;S\;\left(v_{1},\;v_{2}\right)}.$$

To compute ${ℒ}_{\text{Con}}^{D,P}$, a positive pair set $\Psi_{i}^{D,P}$ and a negative pair set $\Omega_{i}^{D,P}$ are first formed. $\Psi_{i}^{D,P}$ contains positive pairs of the domain prototypes $q_{m,k}^{1,t}$ of the first group with respect to the domain features $f_{i}^{D}$ and the remaining domain prototypes of the first and second groups having the same domain label ${\overset{ˆ}{y}}_{m}^{D,t-1}$. It is defined as follows:

(22) $$\begin{array}{ll} \Psi_{i}^{D,P} & =\left\{\left(q_{m,k}^{1,t},f_{i}^{D}\right)|v_{m,k}^{1,t}={\overset{ˆ}{y}}_{m}^{D,t-1}\right\}\\ \; & \cup \left\{\left(q_{m,k}^{1,t},q_{m,r}^{1,t}\right)|v_{m,k}^{1,t}={\overset{ˆ}{y}}_{m}^{D,t-1},v_{m,r}^{1,t}={\overset{ˆ}{y}}_{m}^{D,t-1},k\neq r\right\}\\ \; & \cup \left\{\left(q_{m,k}^{1,t},q_{m,s}^{2,t}\right)|u_{m,k}^{1,t}={\overset{ˆ}{y}}_{m}^{D,t-1},v_{m,s}^{2,t}={\overset{ˆ}{y}}_{m}^{D,t-1}\right\}. \end{array}$$

Meanwhile, $\Omega_{i}^{D,P}$ consists of the negative pairs of the domain prototypes $q_{m,k}^{1,t}$ of the first group with respect to the remaining domain prototypes whose labels are not ${\overset{ˆ}{y}}_{m}^{D,t-1}$, together with the class prototypes. It is defined as follows:

(23) $$\begin{array}{ll} \Omega_{i}^{D,P} & =\left\{\left(q_{m,k}^{1,t},q_{m,r}^{j,t}\right)\right.\\ |v_{m,k}^{1,t} & \left.={\overset{ˆ}{y}}_{m}^{D,t-1},v_{m,r}^{j,t}\neq {\overset{ˆ}{y}}_{m}^{D,t-1}\;\forall \;j\in \{1,\;2\}\right\}\\ \; & \cup \left\{\left(q_{m,k}^{1,t},p_{m,s}^{j,t}\right)|\forall \;j\in \{1,\;2\}\right\}. \end{array}$$

${ℒ}_{\text{Con}}^{D,P}$ is then computed based on $\Psi_{i}^{D,P}$ and $\Omega_{i}^{D,P}$ as follows:

(24) $${ℒ}_{\text{Con}}^{D,P}=-\sum_{x_{i}\in {𝒟}_{m}}log\frac{\sum_{\left(\mu_{1},\mu_{2}\right)\in \Psi_{i}^{D,P}}S\;\left(\mu_{1},\;\mu_{2}\right)}{\sum_{\left(v_{1},v_{2}\right)\in \Psi_{i}^{D,P}\cup \Omega_{i}^{D,P}}S\;\left(v_{1},\;v_{2}\right)}.$$

With dual prototypes and contrastive losses, the HPCL module enforces consistent, representative, and discriminative class features for images of the same class across different domains, thereby mitigating the domain-shift problem.

#### 3) Client Training Loss:

This section introduces the remaining losses used to drive client model training. First, to identify the class of an image $x_{i}$, the CCls make a prediction ${\tilde{y}}_{i}^{C}$ using the class features $f_{i}^{C}$. The class classification loss ${ℒ}_{Cls}^{C}$ is then computed as follows:

(25) $${ℒ}_{Cls}^{C}=-\sum_{x_{i}\in {𝒟}_{m}}y_{i}\;log\;{\tilde{y}}_{i}^{C}.$$

Similarly, the DCls predict the domain label ${\tilde{y}}_{i}^{D}$ using the domain features $f_{i}^{D}$, and the domain classification loss ${ℒ}_{Cls}^{D}$ is computed as follows:

(26) $${ℒ}_{Cls}^{D}=-\sum_{x_{i}\in {𝒟}_{m}}{\overset{ˆ}{y}}_{m}^{D,t-1}\;log\;{\tilde{y}}_{i}^{D}.$$

To regularize the update gradients of the client models, the continual regularization loss ${ℒ}_{R}$ is calculated based on [14] as follows:

(27) $${ℒ}_{R}=\lambda \;{\left\|\left(\theta_{m}^{t}-\theta^{t-1}\right)\right\|}_{2}^{2}$$

where $\theta^{t-1}$ represents the parameters of the global model built in round t-1.

Finally, the client training loss ${ℒ}_{m}^{t}$ is computed as follows:

(28) $${ℒ}_{m}^{t}={ℒ}_{Cls}^{C}+{ℒ}_{Cls}^{D}+{ℒ}_{Con}^{C}+{ℒ}_{Con}^{D}+{ℒ}_{S}+{ℒ}_{R}.$$

#### 4) Client Feature Clustering:

After client training, the class and domain features are clustered to represent the feature distributions of the clients. Clustering is performed based on the neighbor relationship among the features. In particular, for each class c, clustering is performed on the class features of the images belonging to the same class. The centers of the resulting feature clusters are then obtained by averaging the features within each cluster to form a set ${ℋ}_{m}^{C,c}={\left\{h_{m,j}^{C,c}\right\}}_{j=1}^{\gamma_{c}}$ in client ${𝒞}_{m}$, where $\gamma_{c}$ is the number of clusters of class c. Finally, a set ${ℋ}_{m}^{C}$ is formed as follows:

(29) $${ℋ}_{m}^{C}=\left\{{ℋ}_{m}^{C,1},\ldots ,{ℋ}_{m}^{C,c},\ldots ,{ℋ}_{m}^{C,N^{C}}\right\}$$

where $N^{C}$ is the number of classes at the client.

Similarly, clustering is performed on the domain features of client ${𝒞}_{m}$ to obtain a set of cluster centers as follows:

(30) $${ℋ}_{m}^{D}={\left\{h_{m,j}^{D}\right\}}_{j=1}^{K_{m}}$$

where $\kappa_{m}$ is the number of domain feature clusters at client ${𝒞}_{m}$. Since the images of a client belong to the same domain ${\overset{ˆ}{y}}_{m}^{D,t-1}$, the level of ${ℋ}_{m}^{D,d}$ is omitted to simplify the equation.

Finally, the local model, class feature clusters ${ℋ}_{m}^{C}$, and domain feature clusters ${ℋ}_{m}^{D}$ of each client are uploaded to the server for aggregation.

### D. Server Aggregation

To prevent the global model from being biased toward clients with dominant data volumes, the aggregation process is performed with the assistance of the GHA strategy. Different data distributions arising from domain shift lead to different optimal parameter spaces for different domains. To address this issue, the server clusters the client model parameters based on their cosine similarity [68], and clients assigned to the same cluster are considered to belong to the same domain since their local models exhibit similar parameter characteristics. Based on the clustering results, the domain ${\overset{ˆ}{y}}_{m}^{D,t}$ of each client is estimated and assigned for client training in round t+1. A domain model $\theta^{d,t}$ is built for each domain d by aggregating the local models belonging to the same domain using (5). The server then builds the global model $\theta^{t}$ by aggregating all the domain models using (6).

To effectively integrate the different local models, the summation weights $w_{m}^{d}$ and $w^{d}$ of the hierarchical aggregation process are adjusted in accordance with the GMA strategy, as described next. After model aggregation, the server calculates the global class prototypes and global domain prototypes using the feature clusters ${ℋ}_{m}^{C}$ and ${ℋ}_{m}^{D}$ received from the clients. Finally, the global model $\theta^{t}$, global prototypes ${𝒫}^{t}$ and ${𝒬}^{t}$, and domain labels ${\overset{ˆ}{y}}_{m}^{D,t}$ are dispatched to the clients for FL training in the next round.

#### 1) GMA:

Due to the domain-shift problem, the parameters of the local models are diverse. When aggregating the local models, the summation weights of the models play important roles in determining the quality of the global model. However, if the aggregation weights are established solely on the client data volume, the global model may become biased toward the clients with dominant data distributions. To mitigate this effect and more effectively aggregate the different local models, the difference between the local model and the global model in round t-1 is incorporated into the weighting strategy. This difference reflects the gradient update direction of each client and is used to adaptively determine the aggregation weights.

The gradient information can be characterized by both its angle and magnitude. If a local model has large gradients in both angle and magnitude with respect to the global model constructed in the previous round, this indicates that the global model does not adequately fit the private data, and thus, substantial parameter updates of the local model occur during client training. Such a model, therefore, contains additional information not captured in the previous global model and should thus receive a higher aggregation weight. Consequently, in addition to the amount of data possessed by the clients, the summation weights used in the aggregation process also take into account both the angular deviation and magnitude difference between each local model and the previous global model.

Let $\phi_{m}^{t}=1-sim(\theta_{m}^{t*},\theta^{t-1})$ and $\Delta_{m}^{t}=|\theta_{m}^{t*}-\theta^{t-1}|$ represent the angle and magnitude of the local model of client ${𝒞}_{m}$ in round t with respect to the global model in round t-1, respectively. For each local model in domain d, the summation weight $w_{m}^{d}$ used to build the domain models in (5) is based on the angular deviation $\phi_{m}^{t}$, magnitude difference $\Delta_{m}^{t}$, and amount of data at the client as follows:

(31) $$w_{m}^{d}=\frac{N_{m}}{\sum_{\left\{m|{\overset{ˆ}{y}}_{m}^{D,t}=d\right\}}N_{m}}+\alpha \frac{\phi_{m}^{t}\;\cdot \;\Delta_{m}^{t}}{\sum_{\left\{m|{\overset{ˆ}{y}}_{m}^{D,t}=d\right\}}\phi_{m}^{t}\;\cdot \;\Delta_{m}^{t}}$$

where α is a hyperparameter used to adjust the influence of the gradient information. All $w_{m}^{d}$ in domain d is then normalized to a summation of 1. Afterward, the domain model of each domain d is built using (5) based on $w_{m}^{d}$.

Similarly, to aggregate the domain models and build the global model in (6), the summation weight $w^{d}$ for each domain d is computed as follows. Let $N^{d,t}=\sum_{\left\{m|{\overset{ˆ}{y}}_{m}^{D,t}=d\right\}}N_{m}$ be the amount of data in domain d, and $\phi^{d,t}=1-sim(\theta^{d,t},\theta^{t-1})$ and $\Delta^{d,t}=|\theta^{d,t}-\theta^{t-1}|$ represent the angle and magnitude of the difference between the domain model of domain d and the global model built in round t-1, respectively. The summation weight $w^{d}$ for each domain model is computed as follows:

(32) $$w^{d}=\frac{N^{d,t}}{\sum_{d}N^{d,t}}+\alpha \frac{\phi^{d,t}\;\cdot \;\Delta^{d,t}}{\sum_{d}\phi^{d,t}\;\cdot \;\Delta^{d,t}}.$$

All $w^{d}$ values are then normalized to a summation of 1, and the global model is constructed using (6).

#### 2) Global Prototypes Calculation:

After model aggregation, the server derives the global class and domain prototypes using the feature clusters collected from each client, thereby capturing the joint feature distributions of all the domains. As remarked in [3], the class prototypes should not only preserve the diversity of the class features but also serve as consistent anchors to guide feature learning for all the clients. Therefore, the class prototypes of the first group are built by clustering to preserve the variety of the class features, while those of the second group are built based on the average of the feature clusters ${ℋ}_{m}^{C}$ from all the clients to provide a consistent semantic anchor for each class. For each class c, the class feature clusters $\left\{{ℋ}_{m}^{C,c}|{\overset{ˆ}{y}}^{D,t}=d\right\}$ of the clients in domain d are grouped by clustering to form a subset ${𝒢}^{c,d}$ of the class prototypes, where the cluster centers $g_{i}^{c,d}$ serve as prototype representatives as follows:

(33) $${𝒢}^{c,d}={\left\{\left(g_{i}^{c,d},c\right)\right\}}_{i=1}^{\Gamma^{c,d}}$$

where $\Gamma^{c,d}$ is the number of prototypes for class c in domain d. The class prototypes ${𝒫}^{1,t}$ of the first group are then built by taking the union of ${𝒢}^{c,d}$ for all classes and domains, as shown in the following:

(34) $${𝒫}^{1,t}=\underset{c,d}{⋃}{𝒢}^{c,d}.$$

The class prototypes ${𝒫}^{2,t}$ of the second group are built by averaging $g_{i}^{c,d}$ of the same class c as follows:

(35) $${𝒫}^{2,t}=\underset{c}{⋃}\left\{\left({\bar{g}}^{c},c\right)\right\}$$

where ${\bar{g}}^{c}$ is the average of $g_{i}^{c,d}$ and is defined as follows:

(36) $${\bar{g}}^{c}=\frac{1}{\sum_{d}\left|{𝒢}^{c,d}\right|}\sum_{d}\sum_{g_{i}^{c,d}\in {𝒢}^{c,d}}g_{i}^{c,d}.$$

Similarly, for each domain d, the domain feature clusters $\left\{{ℋ}_{m}^{D}|{\overset{ˆ}{y}}^{D,t}=d\right\}$ are grouped via clustering, and the resulting cluster centers $g_{i}^{d}$ form the subset ${𝒢}^{d}$ of domain prototypes as follows:

(37) $${𝒢}^{d}={\left\{\left(g_{i}^{d},d\right)\right\}}_{i=1}^{\Gamma^{d}}$$

where $\Gamma^{d}$ is the number of prototypes in domain d. The domain prototypes ${𝒬}^{1,t}$ and ${𝒬}^{2,t}$ are then built using ${𝒢}^{d}$ as follows:

(38) $${𝒬}^{j,t}=\underset{d}{⋃}{𝒢}^{d}\;\forall \;j\in \{1,\;2\}.$$

The global class prototypes and global domain prototypes are then dispatched to the clients along with the global model for client training in round t+1.

## IV. Experiments

### A. Dataset and Settings

Following recent state-of-the-art work [3], [4], the performance of the proposed method was evaluated on two FL datasets, Digits and Office-Caltech, under both a controlled domain-shift setting in FedHeal [4] and a skewed domain-shift setting in FPL [3].

**Fig. 4.**
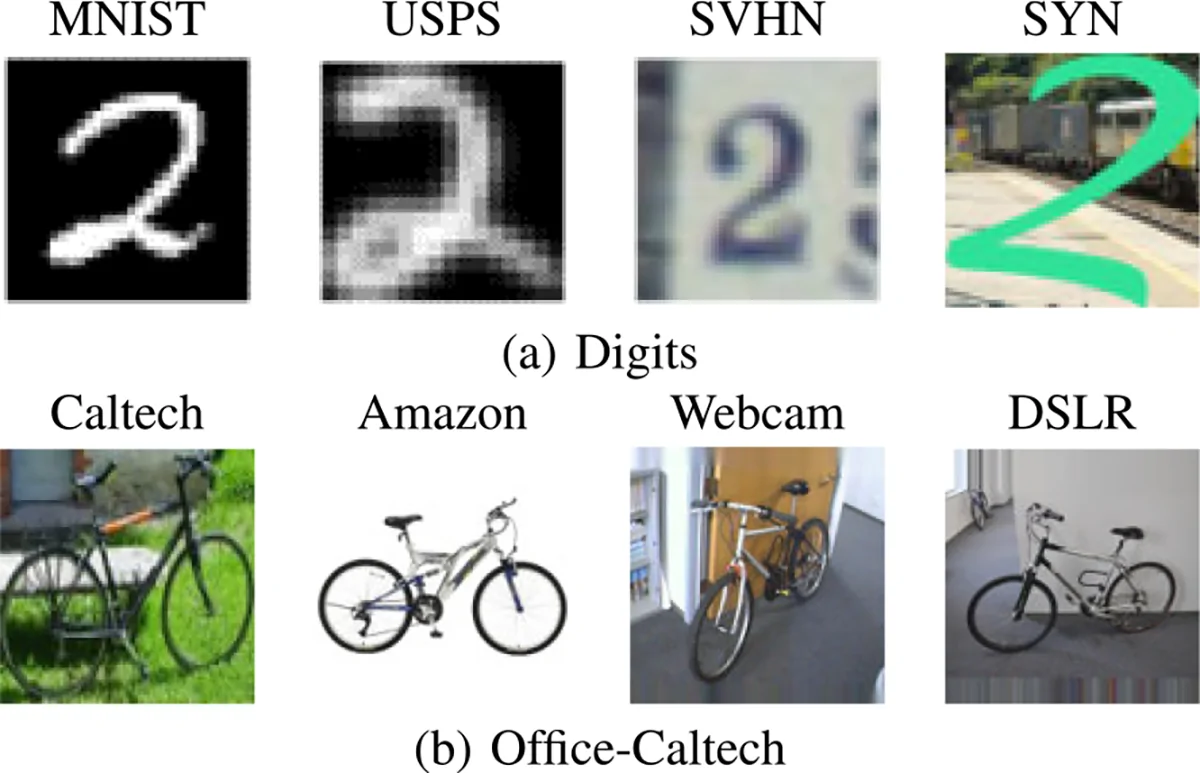
Sample images in (a) Digits dataset and (b) Office-Caltech dataset. Each dataset contains four domains with different styles of appearances, which leads to the domain-shift problem.

As shown in Fig. 4(a), the Digits dataset comprised images of digits 0–9 acquired from four different domains: MNIST [69], USPS [70], SVHN [71], and SYN [72]. Meanwhile, the Office-Caltech dataset consisted of ten overlapping classes of office items from Caltech256 [73] and Office31 [74]. As illustrated in Fig. 4(b), the images within the dataset were distributed across four domains: Caltech, Amazon, Webcam, and DSLR.

To simulate the domain-shift problem, each client was randomly assigned to a domain, and each client randomly sampled the images from the assigned domain.

In the controlled domain-shift setting [4], each domain was assigned to the same number of clients. The controlled setting aimed to evaluate the ability of the proposed FL framework to mitigate the domain-shift problem under controlled conditions.

In contrast, in the skewed domain-shift setting [3], each domain was randomly assigned to an unequal number of clients. That is, some domains were assigned to more clients, while others were assigned to fewer clients. In this case, the domains with larger client populations tended to dominate the model aggregation process, further intensifying the domain-shift problem. As a result, the skewed setting better reflected real-world FL deployments, in which the data distributions are naturally imbalanced. The details of the two settings are described as follows.

In the controlled domain-shift setting [4], both datasets were trained for 200 communication rounds. A total of 20 clients were used, evenly distributed across the four domains, and each client performed 10 local training epochs per round. For the Digits dataset, each client randomly sampled 1% of the data from the target domain, and the batch size was set to 64. In contrast, the Office-Caltech clients randomly sampled 10% of the data from the target domain, and the batch size was set to 16. In the skewed domain-shift setting [3], 20 clients were randomly assigned to different domains for the Digits dataset, and each client randomly selected 1% of the data from the target domain. For the Office-Caltech dataset, ten clients were randomly assigned to different domains, and each client randomly selected 20% of the data from the target domain. For both datasets, training proceeded for 100 communication rounds, and each client performed 10 local training epochs per round with a batch size of 64.

The implementation details are presented in the Supplementary Material.

### B. Ablation Study

The experiments commenced by investigating the effectiveness of each module in the proposed FL framework. Evaluations were performed on both datasets (Digits and Office-Caltech) under the skewed domain-shift setting [3]. Table I shows the ablation study results for each module. As shown in the first row, the baseline model retrained using FedAvg [1] was severely affected by domain shift and achieved a poor performance on both the SYN images of the Digits dataset and the Webcam and DSLR images of the Office-Caltech dataset. Fig. 4 shows that the appearance of the images in these domains differs substantially from those in the other domains, which leads to a more pronounced domain-shift effect and consequently a degraded performance. Referring to the second row in Table I, the continual loss helped constrain the local model gradients and thus slightly improved the overall performance. However, the domain-shift problem persisted. By separating the class and domain features using the proposed CGFS module, the performance of the proposed FL framework for the Webcam and DSLR images on the Office-Caltech dataset significantly improved, as shown in the third row of the table. However, possibly due to the substantial appearance gap between the SYN images and the other domains on the Digits dataset, the CGFS module provided only limited benefit in the SYN domain. When the HPCL module was further introduced, it not only guided the model to learn more consistent features of the same class but also improved the feature discriminability between different classes, as shown in the fourth row of Table I. Therefore, the proposed FL framework significantly improved the FL performance on the SVHN and SYN domains of the Digits dataset, as well as on the DSLR domain of the Office-Caltech dataset. Finally, the proposed GHA strategy helped the server conduct more effective aggregation of the local models from different domains, leading to further performance improvements on the challenging SYN domain of the Digits dataset and Webcam domain of the Office-Caltech dataset, as shown in the last row of Table I.

It is worth noting that GHA aims to prevent the global model from being biased toward certain domains. Therefore, after applying GHA, some domains that previously exhibited a weaker performance showed improvement, while others that were originally favored by aggregation experienced slight performance reductions due to a more balanced contribution of the different domains. Similar observations were also reported in [4]. Overall, each of the proposed modules helped alleviate the domain-shift problem in FL, resulting in improved performance in previously underperforming domains and stronger global accuracy.

Further comparisons of alternative design configurations are available in the Supplementary Material.

### C. Comparison With State-of-the-Art Methods

This section compares the performance of the proposed FL method with various state-of-the-art methods under both a controlled domain-shift setting [4] and a skewed domain-shift setting [3]. For the methods marked with an asterisk (*) in Tables II and III, the reported performance was obtained from FL models trained using their official implementations. The performance of the other methods is reported in [3] and [4].

Table II shows the performance of the various methods when applied to the Digits dataset and Office-Caltech dataset under the controlled domain-shift setting [4]. The best-performing method is highlighted in bold, and the second-best method is underlined. The table is divided into three sections. The performance of the nonprototype-based methods is shown in the first section, while that of the prototype-based methods [3], [11], [12], [13], [56] is presented in the second section. The performance of the proposed method is reported in the third section.

Due to the domain-shift problem, most of the methods performed poorly on domains whose images differed substantially from those of the other domains in the dataset, such as SYN in Digits and Webcam in Office-Caltech. By introducing prototypes to represent the features across clients, FedProto [11] achieved a slight performance improvement compared to the FedAvg baseline. However, the substantial visual differences across domains resulted in heterogeneous feature representations, and the class prototypes obtained by simple feature averaging therefore failed to accurately capture the true class distributions. In addition, simply aligning the features to the prototypes was ineffective in separating the different classes.

To enhance the separation among prototypes of different classes, FedTGP [13] performed contrastive learning on the server. However, it struggled to learn universal representations for different domains and thus performed poorly on domains such as SYN and Caltech. By using cluster prototypes, FPL [3] better captured the domain-specific feature distributions. This then enabled the local models to more effectively learn the object characteristics through contrastive learning with prototypes. Therefore, FPL [3] greatly improved the performance compared to other methods and achieved the second-best performance in both datasets. In the proposed framework, the domain features were disentangled from the class features by the CGFS module, and the model was guided to learn consistent features of the same class by the HPCL module. In addition, the GHA strategy improved the ability of the server to effectively aggregate information from different domains. As a result, the proposed method achieved the best overall performance on both datasets.

Table III compares the performance of the various methods in the two datasets under the skewed domain-shift setting [3]. Consistent with the results in Table II, most of the methods performed poorly on the SYN, Webcam, and DSLR images due to the domain-shift problem. However, through the guidance of feature prototypes, the prototype-based methods [3], [11], [12] greatly improved the performance in the SYN, Webcam, and DSLR images while maintaining the model performance in the other domains. Thus, these methods showed better performance compared to the other methods, with FPL [3] achieving the second-best overall performance.

Beyond leveraging feature prototypes in the HPCL module, the proposed framework explicitly disentangled the class and domain features through the CGFS module and incorporated effective hierarchical aggregation via GHA to reduce the aggregation bias. As a result, it achieved the best overall performance on both datasets under both settings. Regarding the complexity, the proposed method has a similar complexity to that of the other prototype-based methods, albeit with a slightly higher server complexity due to the hierarchical model aggregation. More detailed analysis is presented in the Supplementary Material. These comprehensive comparisons against state-of-the-art methods clearly demonstrate the superiority of the proposed FL framework under heterogeneous domain conditions.

### D. Visualization

**Fig. 5.**
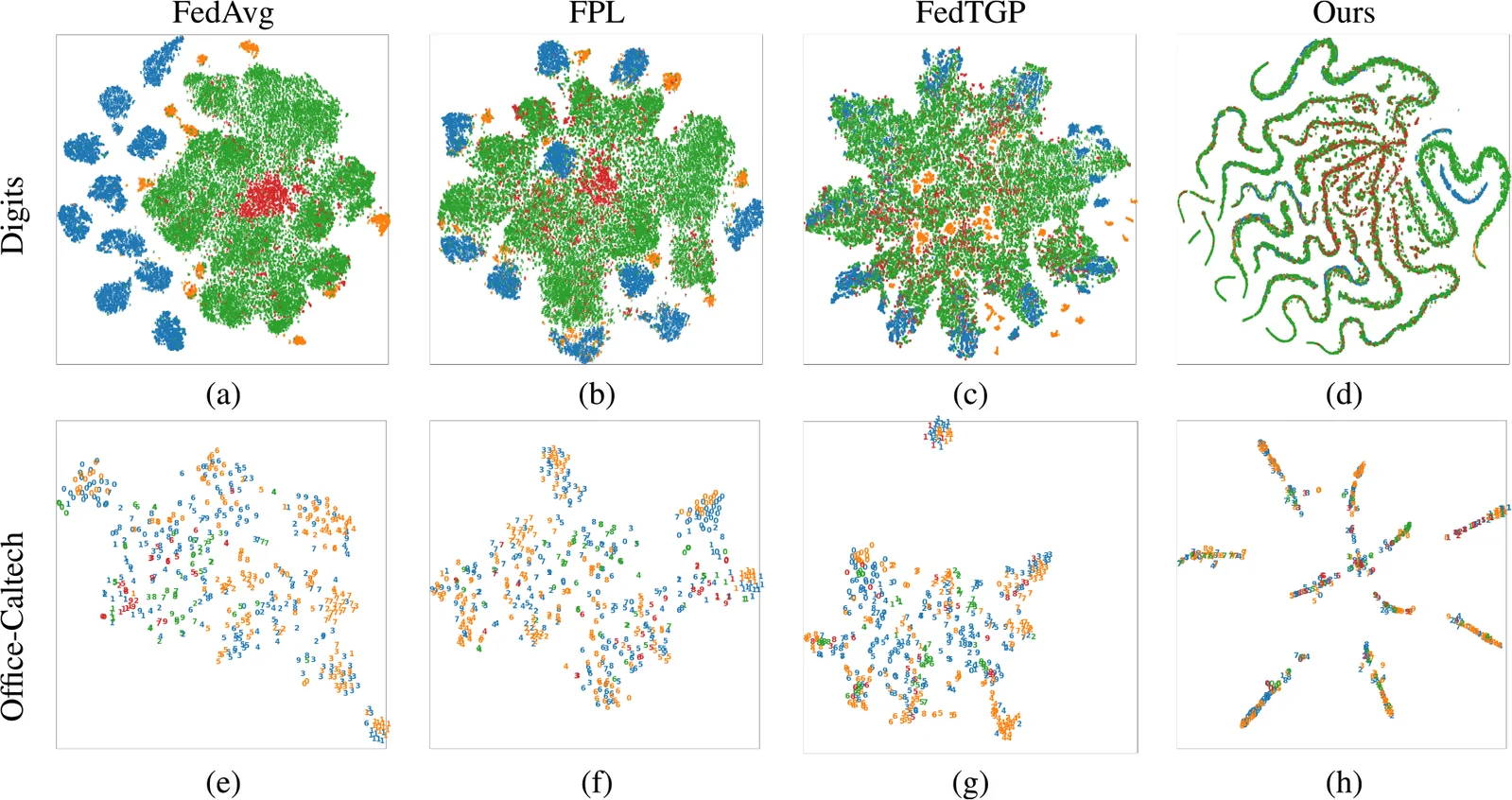
t-SNE visualization results with different colors representing different domains. (a) FedAvg on Digits dataset. (b) FPL on Digits dataset. (c) FedTGP on Digits dataset. (d) Proposed method on Digits dataset. (e) FedAvg on Office-Caltech dataset. (f) FPL on Office-Caltech dataset. (g) FedTGP on Office-Caltech dataset. (h) Proposed method on Office-Caltech dataset.

Fig. 5 presents t-SNE [75] visualizations of the feature distributions learned by FedAvg [1], FPL [3], FedTGP [13], and the proposed method on the Digits and Office-Caltech datasets across different domains. Each point represents the class feature of a testing image from the target dataset, and the color of the point represents the domain of the image. As shown in Fig. 5(a), the FedAvg model separated the features from different domains into distinct regions. This indicates that the domain identity can be inferred directly from the class features. However, it also implies that the class features are affected by domain shift and are determined mainly by domain information rather than class information. Similarly, because prototype-based methods align features solely based on class information, a noticeable separation persists among points of different colors in Fig. 5(b) and (c) for FPL [3] and FedTGP [13], respectively. In contrast, the proposed method separates domain-related information from class-discriminative information through the CGFS module and further guides feature alignment using the HPCL module. Meanwhile, the GHA strategy enables the server to effectively aggregate heterogeneous local models across domains. As a result, class features from different domains become well mixed, making it difficult to infer domain identity from their distributions. This indicates that the learned class features contain minimal domain-specific information, demonstrating the successful disentanglement of the class and domain features, as illustrated in Fig. 5(d). Similar tendencies can be observed in Fig. 5(e)–(h), where the proposed method again achieves superior cross-domain feature alignment compared with the competing methods.

**Fig. 6.**
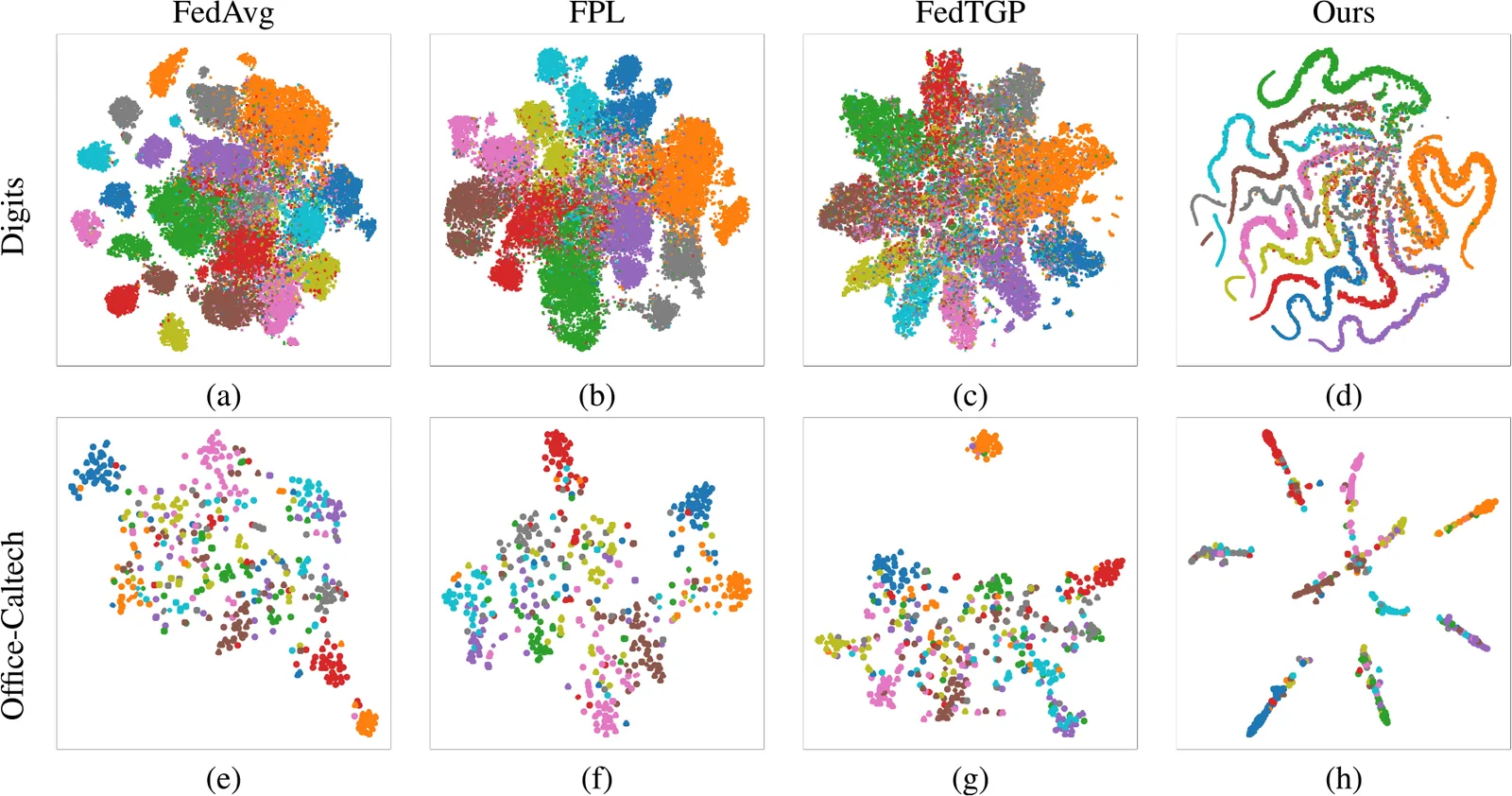
t-SNE visualization results with different colors representing different classes. (a) FedAvg on Digits dataset. (b) FPL on Digits dataset. (c) FedTGP on Digits dataset. (d) Proposed method on Digits dataset. (e) FedAvg on Office-Caltech dataset. (f) FPL on Office-Caltech dataset. (g) FedTGP on Office-Caltech dataset. (h) Proposed method on Office-Caltech dataset.

Fig. 6 shows the t-SNE [75] feature visualization results learned by four methods across different classes. In each figure, the points represent the class features of a testing image selected from the target dataset, and the point colors indicate the class label of the image. As shown in Fig. 6(a), for the FedAvg model, points of the same color are separated into distinct regions. This indicates that the class features of samples from the same class but different domains are inconsistent, reflecting strong domain dependency. Consequently, the learned representations encode domain-specific characteristics rather than purely class-discriminative information. By leveraging class prototypes to guide feature learning, prototype-based methods such as FPL [3] and FedTGP [13] achieve a better alignment of points with the same class, as shown in Fig. 6(b) and (c). However, the learned representations still retain noticeable domain-specific characteristics. That is, some points belonging to the same class remain split across multiple regions, while points from different classes occasionally overlap, indicating an incomplete separation of the class information from the domain information. By contrast, the proposed method explicitly disentangles the class and domain representations through the CGFS and HPCL modules, while the GHA strategy enables the server to effectively integrate information across domains. As a result, the learned class features for images belonging to the same class across different domains become highly consistent, while those of different classes remain clearly separate, as shown in Fig. 6(d). Similar observations can be made in Fig. 6(e)–(h) for the three compared methods and the proposed method in the Office-Caltech dataset. Overall, the t-SNE visualizations demonstrate that the proposed method learns more compact and discriminative class features than FedAvg and prototype-based methods, ultimately contributing to substantial performance improvements in both datasets.

## V. Conclusion

This article presents a class- and domain-aware framework designed to mitigate the domain-shift problem in FL settings. During client training, two newly proposed modules (CGFS and HPCL) are used to explicitly disentangle and learn the class and domain features separately, thereby reducing feature inconsistency across the clients. At the server side, a GHA strategy is employed to facilitate an effective aggregation of the local models by accounting for the client data volumes and domain differences. Extensive experiments were performed on two benchmark datasets. The average accuracy of the proposed method was 0.8% higher than the second-best method on the Digits dataset and 3% higher than the second-best method on the Office-Caltech dataset under the controlled setting. Moreover, in the more challenging skewed setting, the proposed method achieved 1.1% and 1.8% improvements compared to the second-best method on the Digits dataset and Office-Caltech dataset, respectively. Such results show that the proposed method consistently outperforms state-of-the-art approaches under both controlled and skewed domain-shift settings.

There are two promising directions for future work. First, to further reduce the computational overhead of the hierarchical model aggregation process, alternative and more efficient representations of model discrepancy, beyond the cosine similarity of the local model parameters adopted in the present study, will be investigated. Second, because FL aggregates models between clients without sharing data, it is well-suited to train AI models between hospitals due to its inherent privacy-preserving characteristics. However, domain shift commonly occurs in real clinical environments because medical data (e.g., CT or pathology images) are acquired using heterogeneous imaging protocols and devices between hospitals. Therefore, the proposed framework will be specifically adapted to clinical scenarios to enable the development of more robust and reliable federated models for CT images or federated models for pathology images in clinical deployment.

## Supplementary Material

supp1-3658584

This article has supplementary downloadable material available at https://doi.org/10.1109/TNNLS.2026.3658584, provided by the authors.

## Figures and Tables

**TABLE I:** Ablation Study Results on Digits Dataset and Office-Caltech Dataset

${ℒ}_{\mathrm{cont}}$	CGFS	HPCL	GHA	Digits					Office-Caltech				

				MNIST	USPS	SVHN	SYN	Average	Caltech	Amazon	Webcam	DSLR	Average

				97.80	92.87	73.25	40.05	75.99	61.16	75.26	44.83	30.00	52.81
V				97.66	92.13	73.07	41.49	76.09	61.16	75.26	46.55	30.00	53.24
V	V			98.10	**92.92**	74.61	41.39	76.76	63.39	75.79	60.34	36.67	59.05
V	V	V		**98.32**	92.48	79.89	53.48	81.04	**65.18**	**77.89**	53.45	**53.33**	62.46
V	V	V	V	98.17	92.23	**83.16**	**63.43**	**84.25**	63.84	71.05	**65.52**	**53.33**	**63.44**

**TABLE II:** Comparison of Competing Methods on Digits Dataset and Office-Caltech Dataset Under Controlled Domain-Shift Setting [4]

Method	Digit					Office-Caltech				

	MNIST	USPS	SVHN	SYN	Average	Caltech	Amazon	Webcam	DSLR	Average

FedAvg [1]	89.84	93.25	79.54	41.35	76.00	58.57	72.63	45.52	56.67	58.35
FedProx [14]	90.27	93.93	80.04	42.82	76.76	60.27	69.90	45.52	58.00	58.42
Scaffold [15]	94.15	94.44	76.87	44.22	77.42	59.55	69.37	46.21	59.33	58.62
MOON [16]	90.46	92.65	80.48	40.58	76.04	60.63	74.00	46.90	59.33	60.21
FedDyn [18]	91.23	92.36	80.15	41.55	76.32	59.20	71.16	48.62	62.00	60.24
FedAvg + AFL [22]	90.59	**95.83**	75.13	44.42	76.49	57.50	64.21	48.28	65.37	58.83
FedAvg + q-FFL [23]	91.44	94.10	76.33	44.48	76.59	53.39	60.00	51.72	64.01	57.28
Ditto [40]	90.59	92.98	79.20	41.89	76.16	56.25	58.00	63.45	70.00	61.92
FedFV [7]	91.76	94.70	77.26	44.14	76.97	55.36	62.95	60.00	**71.33**	62.41
FedAvg + FedHEAL [4]	90.27	95.69	79.94	46.45	78.09	59.28	67.90	66.21	66.00	64.85
FedAvg + FedRDN* [36]	97.29	93.97	80.63	42.64	78.63	64.29	76.32	68.97	60.00	67.40
FedAWA* [27]	95.98	94.27	79.46	47.81	79.38	58.48	67.37	70.69	53.33	62.47

FedProto [11]	89.99	92.90	81.09	40.93	76.23	62.23	71.48	60.34	42.67	59.18
FedTGP* [13]	96.94	76.91	69.95	12.34	64.04	28.66	44.95	51.03	59.33	46.00
FPL* [3]	**97.98**	93.72	86.12	46.92	81.19	**64.73**	78.42	65.52	60.00	67.17
FedProc [56]	91.86	91.16	78.54	39.87	75.36	55.98	60.21	46.90	46.00	52.27
FedPLVM* [12]	96.21	86.40	85.81	37.06	76.37	54.46	70.53	58.62	53.33	59.24

Ours	97.44	94.17	**86.75**	**49.80**	**82.04**	**64.73**	**83.16**	**74.14**	60.00	**70.51**

**TABLE III:** Comparisons of Competing Methods on Digits Dataset and Office-Caltech Dataset Under Skewed Domain-Shift Setting [3]

Method	Digit					Office-Caltech				

	MNIST	USPS	SVHN	SYN	Average	Caltech	Amazon	Webcam	DSLR	Average

FedAvg [1]	98.14	90.85	76.56	55.01	80.14	60.15	75.44	45.86	36.00	54.36
FedProx [14]	98.11	90.24	77.01	56.66	80.50	60.21	77.44	48.62	37.33	55.90
MOON [16]	97.44	92.15	77.62	38.79	76.50	56.19	71.54	41.04	30.22	49.74
FedDyn [18]	98.01	91.00	78.95	54.22	80.54	61.64	75.54	48.28	35.56	55.25
FedOPT [17]	96.23	91.80	73.03	57.85	79.72	56.31	56.74	63.33	48.89	56.31
FedAvg + FedRDN* [36]	97.25	91.63	71.79	40.55	75.31	58.48	63.16	41.38	53.33	54.09
FedAWA* [27]	97.08	93.37	74.37	50.15	78.74	57.14	53.68	63.79	40.00	53.65

FedProto [11]	98.30	92.44	80.35	53.58	81.16	64.02	**79.37**	50.17	40.33	58.47
FedTGP* [13]	95.96	75.81	65.41	12.13	62.33	42.56	68.95	63.79	**67.5**	60.70
FPL [3]	**98.31**	**92.71**	80.27	61.20	83.12	63.39	79.26	55.86	48.00	61.63
FedProc [56]	97.86	88.99	78.90	45.84	77.89	58.07	73.65	42.76	30.22	51.17
FedPLVM* [12]	96.81	89.29	82.17	40.25	77.13	**64.73**	78.42	56.90	43.33	60.85

Ours	98.17	92.23	**83.16**	**63.43**	**84.25**	63.84	71.05	**65.52**	53.33	**63.44**

## References

[1] McMahan B, Moore E, Ramage D, Hampson S, and Arcas BAY, “Communication-efficient learning of deep networks from decentralized data,” in Proc. 20th Int. Conf. Artif. Intell. Statist., vol. 54, Singh A and Zhu J, Eds., Fort Lauderdale, FL, USA, 2017, pp. 1273–1282.

[2] Li T, Sahu AK, Talwalkar A, and Smith V, “Federated learning: Challenges, methods, and future directions,” IEEE Signal Process. Mag., vol. 37, no. 3, pp. 50–60, May 2020.

[3] Huang W, Ye M, Shi Z, Li H, and Du B, “Rethinking federated learning with domain shift: A prototype view,” in Proc. IEEE/CVF Conf. Comput. Vis. Pattern Recognit. (CVPR), Jun. 2023, pp. 16312–16322.

[4] Chen Y, Huang W, and Ye M, “Fair federated learning under domain skew with local consistency and domain diversity,” in Proc. IEEE/CVF Conf. Comput. Vis. Pattern Recognit. (CVPR), Jun. 2024, pp. 12077–12086.

[5] Zhou T, Yuan Y, Wang B, and Konukoglu E, “Federated feature augmentation and alignment,” IEEE Trans. Pattern Anal. Mach. Intell., vol. 46, no. 12, pp. 11119–11135, Dec. 2024.

[6] Wang Z, Wang Z, Wang Z, Fan X, and Wang C, “Federated learning with domain shift eraser,” in Proc. IEEE/CVF Conf. Comput. Vis. Pattern Recognit. (CVPR), Jun. 2025, pp. 4978–4987.

[7] Wang Z, Fan X, Qi J, Wen C, Wang C, and Yu R, “Federated learning with fair averaging,” in Proc. Int. Joint Conf. Artif. Intell., 2021, pp. 1615–1623.

[8] Lim J and Kim Y, “Cross-domain semantic segmentation on inconsistent taxonomy using VLMs,” in Proc. Euro. Conf. Comput. Vis., 2024, pp. 18–35.

[9] Tan Z, Yang X, and Huang K, “Rethinking multi-domain generalization with a general learning objective,” in Proc. IEEE/CVF Conf. Comput. Vis. Pattern Recognit. (CVPR), Jun. 2024, pp. 23512–23522.

[10] Zbontar J, Jing L, Misra I, LeCun Y, and Deny S, “Barlow twins: Self-supervised learning via redundancy reduction,” in Proc. Int. Conf. Mach. Learn., 2021, pp. 12310–12320.

[11] Tan Y , “FedProto: Federated prototype learning across heterogeneous clients,” in Proc. 36th AAAI Conf. Artif. Intell., 2022, pp. 8432–8440.

[12] Wang L, Bian J, Zhang L, Chen C, and Xu J, “Taming cross-domain representation variance in federated prototype learning with heterogeneous data domains,” in Proc. Adv. Neural Inf. Process. Syst., 2024, pp. 88348–88372.

[13] Zhang J, Liu Y, Hua Y, and Cao J, “FedTGP: Trainable global prototypes with adaptive-margin-enhanced contrastive learning for data and model heterogeneity in federated learning,” in Proc. AAAI Conf. Artif. Intell., 2024, vol. 38, no. 15, pp. 16768–16776.

[14] Li T, Sahu AK, Zaheer M, Sanjabi M, Talwalkar A, and Smith V, “Federated optimization in heterogeneous networks,” in Proc. Mach. Learn. Syst., 2018, pp. 429–450.

[15] Karimireddy SP , “SCAFFOLD: Stochastic controlled averaging for federated learning,” in Proc. Int. Conf. Mach. Learn., 2019, pp. 5132–5143.

[16] Li Q, He B, and Song D, “Model-contrastive federated learning,” in Proc. IEEE/CVF Conf. Comput. Vis. Pattern Recognit. (CVPR), Jun. 2021, pp. 10708–10717.

[17] Reddi SJ , “Adaptive federated optimization,” in Proc. Int. Conf. Learn. Represent., 2021, pp. 1–12.

[18] Acar DAE, Zhao Y, Navarro RM, Mattina M, Whatmough PN, and Saligrama V, “Federated learning based on dynamic regularization,” in Proc. Int. Conf. Learn. Represent., 2021, pp. 1–12.

[19] Luo K, Li X, Lan Y, and Gao M, “GradMA: A gradient-memory-based accelerated federated learning with alleviated catastrophic forgetting,” in Proc. IEEE/CVF Conf. Comput. Vis. Pattern Recognit. (CVPR), Jun. 2023, pp. 3708–3717.

[20] Son HM, Kim M-H, Chung T-M, Huang C, and Liu X, “FedUV: Uniformity and variance for heterogeneous federated learning,” in Proc. IEEE/CVF Conf. Comput. Vis. Pattern Recognit. (CVPR), Jun. 2024, pp. 5863–5872.

[21] Wei Y and Han Y, “Multi-source collaborative gradient discrepancy minimization for federated domain generalization,” in Proc. AAAI Conf. Artif. Intell., 2024, vol. 38, no. 14, pp. 15805–15813.

[22] Mohri M, Sivek G, and Suresh AT, “Agnostic federated learning,” in Proc. Int. Conf. Mach. Learn., 2019, pp. 4615–4625.

[23] Li T, Sanjabi M, Beirami A, and Smith V, “Fair resource allocation in federated learning,” in Proc. Int. Conf. Learn. Represent., 2020, pp. 1–13.

[24] Hosseini SM, Sikaroudi M, Babaie M, and Tizhoosh HR, “Proportionally fair hospital collaborations in federated learning of histopathology images,” IEEE Trans. Med. Imag., vol. 42, no. 7, pp. 1982–1995, Jul. 2023. 10.1109/TMI.2023.3234450 37018335

[25] Li Z, Lin T, Shang X, and Wu C, “Revisiting weighted aggregation in federated learning with neural networks,” in Proc. Int. Conf. Mach. Learn., 2023, pp. 19767–19788.

[26] Shi C, Li J, Zhao H, Guo D, and Chang Y, “FedLWS: Federated learning with adaptive layer-wise weight shrinking,” in Proc. Int. Conf. Learn. Represent., 2025, pp. 1–13.

[27] Shi C, Zhao H, Zhang B, Zhou M, Guo D, and Chang Y, “FedAWA: Adaptive optimization of aggregation weights in federated learning using client vectors,” in Proc. IEEE/CVF Conf. Comput. Vis. Pattern Recognit. (CVPR), Jun. 2025, pp. 30651–30660.

[28] Farhadkhani S, Guerraoui R, Gupta N, Pinot R, and Stephan JJ, “Byzantine machine learning made easy by resilient averaging of momentums,” in Proc. Int. Conf. Mach. Learn., 2022, pp. 6246–6283.

[29] Allouah Y, Guerraoui R, Gupta N, Jellouli A, Rizk G, and Stephan JJ, “Adaptive gradient clipping for robust federated learning,” in Proc. Int. Conf. Learn. Represent., 2025, pp. 1–14.

[30] Liu Q, Chen C, Qin J, Dou Q, and Heng P-A, “FedDG: Federated domain generalization on medical image segmentation via episodic learning in continuous frequency space,” in Proc. IEEE/CVF Conf. Comput. Vis. Pattern Recognit. (CVPR), Jun. 2021, pp. 1013–1023.

[31] Liu Y, Guo S, Zhang J, Zhan Y, Zhou Q, and Wang Y, “Feature correlation-guided knowledge transfer for federated self-supervised learning,” IEEE Trans. Neural Netw. Learn. Syst., vol. 36, no. 6, pp. 10544–10557, Jun. 2025. 10.1109/TNNLS.2025.3541642 40031710

[32] Hong M, Yun J, Jeon I, and Kim G, “FedAvP: Augment local data via shared policy in federated learning,” in Proc. Adv. Neural Inf. Process. Syst., 2024, pp. 18090–18121.

[33] Yuan H, Hui B, Yang Y, Burlina P, Gong NZ, and Cao Y, “Addressing heterogeneity in federated learning via distributional transformation,” in Proc. Euro. Conf. Comput. Vis., 2022, pp. 179–195.

[34] Yang B, Yuen PC, Zhang Y, and Zeng A, “Allosteric feature collaboration for model-heterogeneous federated learning,” IEEE Trans. Neural Netw. Learn. Syst., vol. 36, no. 2, pp. 3042–3056, Feb. 2025. 10.1109/TNNLS.2023.3344084 38145509

[35] Chen H, Zhu T, Zhou W, and Zhao W, “AFed: Algorithmic fair federated learning,” IEEE Trans. Neural Netw. Learn. Syst., vol. 36, no. 8, pp. 1–13, Aug. 2025. 10.1109/TNNLS.2025.3528012 40031278

[36] Yan Y , “A simple data augmentation for feature distribution skewed federated learning,” in Proc. IEEE/CVF Conf. Comput. Vis. Pattern Recognit. (CVPR), Jun. 2025, pp. 25749–25758.

[37] Yan Y, Tong X, and Wang S, “Clustered federated learning in heterogeneous environment,” IEEE Trans. Neural Netw. Learn. Syst., vol. 35, no. 9, pp. 12796–12809, Sep. 2024. 10.1109/TNNLS.2023.3264740 37079405

[38] Huang H , “Active client selection for clustered federated learning,” IEEE Trans. Neural Netw. Learn. Syst., vol. 35, no. 11, pp. 16424–16438, Nov. 2024. 10.1109/TNNLS.2023.3294295 37506017

[39] Guo Y, Tang X, and Lin T, “Enhancing clustered federated learning: Integration of strategies and improved methodologies,” in Proc. Int. Conf. Learn. Represent., 2025, pp. 1–14.

[40] Li T, Hu S, Beirami A, and Smith V, “Ditto: Fair and robust federated learning through personalization,” in Proc. Int. Conf. Mach. Learn., 2021, pp. 6357–6368.

[41] Yang X, Huang W, and Ye M, “FedAS: Bridging inconsistency in personalized federated learning,” in Proc. IEEE/CVF Conf. Comput. Vis. Pattern Recognit. (CVPR), Jun. 2024, pp. 11986–11995.

[42] Li H , “FedTP: Federated learning by transformer personalization,” IEEE Trans. Neural Netw. Learn. Syst., vol. 35, no. 10, pp. 13426–13440, Oct. 2024. 10.1109/TNNLS.2023.3269062 37220054

[43] Meng L , “Improving global generalization and local personalization for federated learning,” IEEE Trans. Neural Netw. Learn. Syst., vol. 36, no. 1, pp. 76–87, Jan. 2025. 10.1109/TNNLS.2024.3417452 39028598

[44] Zhang Y , “Federated discriminative representation learning for image classification,” IEEE Trans. Neural Netw. Learn. Syst., vol. 36, no. 2, pp. 3204–3217, Feb. 2025. 10.1109/TNNLS.2023.3336957 38055356

[45] Luo J, Chen C, and Wu S, “Mixture of experts made personalized: Federated prompt learning for vision-language models,” in Proc. Int. Conf. Learn. Represent., 2025, pp. 1–14. PMC12904679 41695777

[46] Liu X, Deng Y, Nallanathan A, and Bennis M, “Federated learning and meta learning: Approaches, applications, and directions,” IEEE Commun. Surveys Tuts., vol. 26, no. 1, pp. 571–618, 1st Quart., 2024.

[47] Liu S , “AFM3D: An asynchronous federated meta-learning framework for driver distraction detection,” IEEE Trans. Intell. Transp. Syst., vol. 25, no. 8, pp. 9659–9674, Aug. 2024.

[48] Wang Q, Xu S, Xu R, and Ai B, “A model-prediction-based hierarchical personalized federated learning framework with distributed resource optimization,” IEEE Trans. Commun., vol. 73, no. 12, pp. 14212–14226, Dec. 2025.

[49] You L, Guo Z, Yuen C, Chen CY-C, Zhang Y, and Poor HV, “A framework reforming personalized Internet of Things by federated meta-learning,” Nature Commun., vol. 16, no. 1, p. 3739, Apr. 2025. 10.1038/s41467-025-59217-z PMC12009980 40254640

[50] Li Y, Xu W, Wang H, Qi Y, Guo J, and Li R, “Personalized federated domain-incremental learning based on adaptive knowledge matching,” in Proc. Euro. Conf. Comput. Vis., 2024, pp. 127–144.

[51] Zhang Y, Zhu H, Tan AZ, Yu D, Huang L, and Yu H, “PFedMxF: Personalized federated class-incremental learning with mixture of frequency aggregation,” in Proc. IEEE/CVF Conf. Comput. Vis. Pattern Recognit. (CVPR), Jun. 2025, pp. 30640–30650.

[52] Li Y, Wang H, Qi Y, Liu W, and Li R, “Re-Fed+: A better replay strategy for federated incremental learning,” IEEE Trans. Pattern Anal. Mach. Intell., vol. 47, no. 7, pp. 5489–5500, Jul. 2025. 10.1109/TPAMI.2025.3551732 40095835

[53] Dai Y, Chen Z, Li J, Heinecke S, Sun L, and Xu R, “Tackling data heterogeneity in federated learning with class prototypes,” in Proc. AAAI Conf. Artif. Intell., 2023, vol. 37, no. 6, pp. 7314–7322.

[54] Shi J, Zheng S, Yin X, Lu Y, Xie Y, and Qu Y, “CLIP-guided federated learning on heterogeneity and long-tailed data,” in Proc. AAAI Conf. Artif. Intell., 2024, vol. 38, no. 13, pp. 14955–14963.

[55] Radford A , “Learning transferable visual models from natural language supervision,” in Proc. Int. Conf. Mach. Learn., vol. 139, 2021, pp. 8748–8763.

[56] Mu X , “FedProc: Prototypical contrastive federated learning on non-IID data,” Future Gener. Comput. Syst., vol. 143, pp. 93–104, Jun. 2023.

[57] Wan G, Huang W, and Ye M, “Federated graph learning under domain shift with generalizable prototypes,” in Proc. AAAI Conf. Artif. Intell., 2024, vol. 38, no. 14, pp. 15429–15437.

[58] Han J, Shan C, and Han Y, “FedGMKD: An efficient prototype federated learning framework through knowledge distillation and discrepancy-aware aggregation,” in Proc. Adv. Neural Inf. Process. Syst., 2024, pp. 118326–118356.

[59] Fu L, Huang S, Lai Y, Liao T, Zhang C, and Chen C, “Beyond federated prototype learning: Learnable semantic anchors with hyperspherical contrast for domain-skewed data,” in Proc. AAAI Conf. Artif. Intell., 2025, vol. 39, no. 16, pp. 16648–16656.

[60] Zhou Y , “FedSA: A unified representation learning via semantic anchors for prototype-based federated learning,” in Proc. AAAI Conf. Artif. Intell., 2025, vol. 39, no. 21, pp. 23009–23017.

[61] Guo S, Wang H, Lin S, Kou Z, and Geng X, “Addressing skewed heterogeneity via federated prototype rectification with personalization,” IEEE Trans. Neural Netw. Learn. Syst., vol. 36, no. 5, pp. 8442–8454, May 2025. 10.1109/TNNLS.2024.3438281 39190523

[62] An X , “Federated learning with only positive labels by exploring label correlations,” IEEE Trans. Neural Netw. Learn. Syst., vol. 36, no. 4, pp. 7651–7665, Apr. 2025. 10.1109/TNNLS.2024.3396875 38743541

[63] Arunan A, Qin Y, Li X, and Yuen C, “Intelligently augmented contrastive tensor factorization: Empowering multi-dimensional time series classification in low-data environments,” Expert Syst. Appl., vol. 287, Aug. 2025, Art. no. 127889.

[64] Yang W , “FedMPS: Federated learning in a synergy of multi-level prototype-based contrastive learning and soft label generation,” IEEE Trans. Neural Netw. Learn. Syst., pp. 1–14, 2025. 10.1109/TNNLS.2025.3611832 41052182

[65] Chen T, Kornblith S, Norouzi M, and Hinton G, “A simple framework for contrastive learning of visual representations,” in Proc. Int. Conf. Mach. Learn., 2020, pp. 1597–1607.

[66] Chiou C-Y, Lee K-T, Huang C-R, and Chung P-C, “ADMM-SRNet: Alternating direction method of multipliers based sparse representation network for one-class classification,” IEEE Trans. Image Process., vol. 32, pp. 2843–2856, 2023. 10.1109/TIP.2023.3274488 37171924

[67] Chen Y-T, Chiou C-Y, and Huang C-R, “Interactive augmentations, features, and parameters for contrastive learning [AI-eXplained],” IEEE Comput. Intell. Mag., vol. 19, no. 1, pp. 79–80, Feb. 2024.

[68] Sarfraz S, Sharma V, and Stiefelhagen R, “Efficient parameter-free clustering using first neighbor relations,” in Proc. IEEE/CVF Conf. Comput. Vis. Pattern Recognit. (CVPR), Jun. 2019, pp. 8934–8943.

[69] Lecun Y, Bottou L, Bengio Y, and Haffner P, “Gradient-based learning applied to document recognition,” Proc. IEEE, vol. 86, no. 11, pp. 2278–2324, 1998.

[70] Hull JJ, “A database for handwritten text recognition research,” IEEE Trans. Pattern Anal. Mach. Intell., vol. 16, no. 5, pp. 550–554, May 1994.

[71] Netzer Y , “Reading digits in natural images with unsupervised feature learning,” in Proc. NIPS Workshop Deep Learn. Unsupervised Feature Learn., 2024, p. 4.

[72] Roy P, Ghosh S, Bhattacharya S, and Pal U, “Effects of degradations on deep neural network architectures,” 2018, arXiv:1807.10108.

[73] Griffin G, Holub A, and Perona P, “Caltech-256 object category dataset,” California Institute Technology Pasadena, Pasadena, CA, USA, Tech. Rep. 7694, 2007.

[74] Saenko K, Kulis B, Fritz M, and Darrell T, “Adapting visual category models to new domains,” in Proc. Eur. Conf. Comput. Vis. (ECCV), 2010, pp. 213–226.

[75] Van der Maaten L and Hinton GE, “Visualizing data using t-SNE,” J. Mach. Learn. Res., vol. 9, no. 86, pp. 2579–2605, 2008.

