# Supplementary material for: Using Class and Domain Information to Address Domain Shift in Federated Learning
Source: IEEE Trans Neural Netw Learn Syst. Author manuscript; Available in PMC 2026 Sep 23. (PMC13598942; doi:10.1109/TNNLS.2026.3658584)
Supplement: Supplementary file 1 [file NIHMS2208468-supplement-supp1-3658584.pdf]

Using Class and Domain Information to Address Domain Shift in Federated Learning

Supplementary Material

Chien-Yu Chiou, *Member, IEEE,*, Chun-Rong Huang, *Senior Member, IEEE*, Lawrence L. Latour, Yang C. Fann, and Pau-Choo Chung, *Fellow, IEEE*

I. Implementation Details

The proposed framework was implemented using PyTorch v2.1.1. Following the same setting as that used in [1], [2], a ResNet-10 [3] encoder backbone was employed without architectural modification, thereby ensuring that performance differences were the result solely of the FL learning strategy rather than changes to the feature extractor. Optimization was performed using the SGD optimizer with a learning rate of 0.01, a weight decay of $1e-5$, and a momentum of 0.9. Parameter λ of the continual loss was set to 0.01, and τ was set to 0.5 for both the class contrastive loss and the domain contrastive loss. The value of α used in Eq. (31) and Eq. (32) was empirically set as 1 for the Digits dataset and 0.5 for the Office-Caltech dataset. Finally, clustering was performed using FINCH [4], which is a parameter-free clustering algorithm suitable for FL and is recommended by [1].

II. More Detailed Ablation Study

Table I shows the effectiveness of the proposed CGFS module with the inverse gating function $I_v(\cdot)$. The first row shows the results of the CGFS module without $I_v(\cdot)$, where $I_v(\cdot)$ suppresses the responses of the class features based on the domain features, and vice versa. In other words, it enforces the separation of the class and domain features. Without $I_v(\cdot)$, the performance drops significantly for SYN images in the Digits dataset. Similarly, the performance of the Webcam and DSLR images also decreases in the Office-Caltech dataset. These results confirm the importance of $I_v(\cdot)$ in effectively disentangling the class features and domain features in the input images.

Table II shows the effectiveness of the proposed dual-prototype and heterogeneous prototype contrastive losses. The dual-class prototypes $\mathcal{P}_m^t$ and dual-domain prototypes $\mathcal{Q}_m^t$ were constructed from the respective feature clusters to effectively capture the distributions of the class and domain features, respectively. Moreover, to guide the learning of class features, heterogeneous prototype contrastive losses contain the class contrastive loss $\mathcal{L}_{Con}^C$, and domain contrastive loss $\mathcal{L}_{Con}^D$. $\mathcal{L}_{Con}^C$ and $\mathcal{L}_{Con}^D$ were computed based on both class prototypes and domain prototypes.

In the first row of Table II, the dual-class prototypes and dual-domain prototypes are replaced with simple prototypes; that is, the distribution of the class features for each class is represented with a single class prototype computed by taking the average of the corresponding class features. The domain prototype for each domain is constructed in an analogous manner. Compared with the proposed method (shown in the third row), the model performance decreases moderately for the MNIST images and SVHN images of the Digits dataset, and a substantial drop is observed for the SYN images. The performance similarly drops for all four domains in the Office-Caltech dataset.

These findings imply that using only a single averaging-based prototype for each class or domain is insufficient to capture the diversity within the feature distribution. Therefore, the proposed clustering-based dual-class and dual-domain prototypes are essential for effectively representing the complex feature distributions across different classes and domains.

In the second row of Table II, $\mathcal{L}_{Con}^C$ and $\mathcal{L}_{Con}^D$ are replaced by simple contrastive losses; that is, the class features are learned using only class prototypes, and the domain features are learned using only domain prototypes. Compared with the proposed method, the model performance again decreases on both datasets, particularly for the SYN images in the Digits dataset and DSLR images in the Office-Caltech dataset. As the domain prototypes represent the distributions of the domain features of all the clients, the class contrastive loss is not guaranteed to push the class features away from the domain features of other domains if the domain prototypes are not also considered. In the same way, for the domain contrastive loss, it is also necessary to consider the class feature information. Therefore, it is important to consider both the class prototypes and the domain prototypes in $\mathcal{L}_{Con}^C$ and $\mathcal{L}_{Con}^D$.

Table III compares the performance results obtained when using different similarity metrics to cluster the models in the proposed GHA strategy. In particular, besides using the cosine similarity of the model parameters to represent the consistency between models, the table also considers the similarities between the class features and domain features, respectively. The results confirm that, since the model parameters encode the information learned from the client data, the proposed design achieves the best performance, as shown in the third row of the table. Overall, the results of the ablation studies confirm the effectiveness of the proposed method.

III. Discussion

Table IV compares the complexity of the proposed method with that of the FedAvg baseline [5] and several other

TABLE I
VARIANTS OF CGFS ON DIGITS DATASET AND OFFICE-CALTECH DATASET.

Feature Separation	Digits					Office-Caltech				
	MNIST	USPS	SVHN	SYN	Average	Caltech	Amazon	Webcam	DSLR	Average
CGFS without $I_v(\cdot)$	98.02	**92.78**	79.94	48.31	79.76	61.16	70.53	60.34	30.00	55.51
CGFS	**98.17**	92.23	**83.16**	**63.43**	**84.25**	**63.84**	**71.05**	**65.52**	**53.33**	**63.44**

TABLE II
VARIANTS OF PROTOTYPES AND HPCL ON DIGITS DATASET AND OFFICE-CALTECH DATASET.

Prototypes	Prototypes Loss	Digits					Office-Caltech				
		MNIST	USPS	SVHN	SYN	Average	Caltech	Amazon	Webcam	DSLR	Average
Simple Prototypes	HPCL	96.71	92.78	80.77	44.38	78.66	55.80	65.26	60.34	40.00	55.35
Dual Prototypes	Simple Contrastive Loss	98.15	**93.02**	80.66	57.36	82.30	**66.96**	**71.05**	60.34	43.33	60.42
Dual Prototypes	HPCL	**98.17**	92.23	**83.16**	**63.43**	**84.25**	63.84	**71.05**	**65.52**	**53.33**	**63.44**

TABLE III
VARIANTS OF MODEL CLUSTERING SIMILARITY ON DIGITS DATASET AND OFFICE-CALTECH DATASET.

Model Clustering Similarity	Digits					Office-Caltech				
	MNIST	USPS	SVHN	SYN	Average	Caltech10	Amazon	Webcam	DSLR	Average
Class Features	97.58	91.03	**85.15**	61.34	83.78	**64.29**	**74.74**	55.17	50.00	61.05
Domain Features	98.03	**93.22**	81.81	57.41	82.62	62.50	70.00	60.34	**53.33**	61.54
Model Parameters (Ours)	**98.17**	92.23	83.16	**63.43**	**84.25**	63.84	71.05	**65.52**	**53.33**	**63.44**

TABLE IV
COMPLEXITY ANALYSIS FOR ONE FL ROUND. EACH COMPLEXITY IS REPRESENTED BY TWO TERMS. THE FIRST TERM REPRESENTS THE COMPLEXITY RELATED TO THE MODEL, WHILE THE SECOND TERM REPRESENTS THE COMPLEXITY RELATED TO THE PROTOTYPE.

Method	Client Training (Each Client)	Communication (Each Client)	Server Aggregation (Total)
FedAvg [5]	$\mathcal{O}(E_c \cdot N \cdot \lvert\theta\rvert) + 0$	$\mathcal{O}(\lvert\theta\rvert) + 0$	$\mathcal{O}(M \cdot \lvert\theta\rvert) + 0$
FedProto [6]	$\mathcal{O}(E_c \cdot N \cdot (\lvert\theta\rvert + C)) + \mathcal{O}(N \cdot \lvert\theta\rvert)$	$0 + \mathcal{O}(C)$	$0 + \mathcal{O}(C \cdot M)$
FedTGP [7]	$\mathcal{O}(E_c \cdot N \cdot (\lvert\theta\rvert + C)) + \mathcal{O}(N \cdot \lvert\theta\rvert)$	$0 + \mathcal{O}(C)$	$0 + \mathcal{O}(E_s \cdot C \cdot (\vartheta + C \cdot M))$
FPL [1]	$\mathcal{O}(E_c \cdot N \cdot (\lvert\theta\rvert + \lvert\mathcal{P}\rvert)) + \mathcal{O}(N \cdot \lvert\theta\rvert)$	$\mathcal{O}(\lvert\theta\rvert) + \mathcal{O}(C)$	$\mathcal{O}(M \cdot \lvert\theta\rvert) + \mathcal{O}(C \cdot M^2)$
FedProc [8]	$\mathcal{O}(E_c \cdot N \cdot (\lvert\theta\rvert + \lvert\mathcal{P}\rvert)) + \mathcal{O}(N \cdot \lvert\theta\rvert)$	$\mathcal{O}(\lvert\theta\rvert) + \mathcal{O}(C)$	$\mathcal{O}(M \cdot \lvert\theta\rvert) + \mathcal{O}(C \cdot M)$
FedPLVM [9]	$\mathcal{O}(E_c \cdot N \cdot (\lvert\theta\rvert + \lvert\mathcal{P}\rvert)) + \mathcal{O}(N \cdot \lvert\theta\rvert + N^2)$	$\mathcal{O}(\lvert\theta\rvert) + \mathcal{O}(\lvert\mathcal{P}\rvert)$	$\mathcal{O}(M \cdot \lvert\theta\rvert) + \mathcal{O}(C \cdot (M \cdot \frac{\lvert\mathcal{P}\rvert}{C})^2)$
Ours	$\mathcal{O}(E_c \cdot N \cdot (\lvert\theta\rvert + \lvert\mathcal{P}\rvert)) + \mathcal{O}(N \cdot \lvert\theta\rvert + N^2)$	$\mathcal{O}(\lvert\theta\rvert) + \mathcal{O}(\lvert\mathcal{P}\rvert)$	$\mathcal{O}(M^2 \cdot \lvert\theta\rvert) + \mathcal{O}(C \cdot (M \cdot \frac{\lvert\mathcal{P}\rvert}{C})^2)$

M: number of clients, N: number of data, C: number of classes, E_c: client training epoch, $|\theta|$: size of FL models, $|\mathcal{P}|$: number of prototypes, E_s: additional server training epoch, ϑ: size of additional server-side model

prototype-based methods, including FedProto [6], FedTGP [7], FPL [1], FedProc [8], and FedPLVM [9]. Each complexity element in the table contains two terms: one term representing the complexity of the model and the other representing the complexity of the prototype. Since FedAvg [5] does not use prototypes, only the complexity term related to the model is shown. Similarly, FedProto [6] and FedTGP [7] transmit only prototypes to the server, and hence only the complexity term related to prototype communication and aggregation is shown. It is seen in the table that the proposed method has a similar complexity to that of the other prototype-based methods, albeit with a slightly higher server complexity due to the hierarchical model aggregation process.

The proposed method hierarchically aggregates the local models based on the cosine similarity of the model parameters. The aggregating process thus has a complexity of similarity among all clients, which has complexity $\mathcal{O}(M^2|\theta|)$. When the number of clients becomes extremely large, the server aggregation process may therefore incur a significant computational load. Thus, in future work, we aim to develop more efficient and effective strategies for representing information from different domains and for measuring the similarity among local models to reduce the computational complexity of the aggregation process.

REFERENCES

[1] W. Huang, M. Ye, Z. Shi, H. Li, and B. Du, "Rethinking federated learning with domain shift: A prototype view," in *Proc. IEEE Conf. Computer Vision and Pattern Recognition*, 2023, pp. 16 312–16 322.

[2] Y. Chen, W. Huang, and M. Ye, "Fair federated learning under domain skew with local consistency and domain diversity," in *Proc. IEEE Conf. Computer Vision and Pattern Recognition*, 2024, pp. 12 077–12 086.

[3] K. He, X. Zhang, S. Ren, and J. Sun, "Deep residual learning for image recognition," in *Proc. IEEE Conf. Computer Vision and Pattern Recognition*, 2016, pp. 770–778.

[4] S. Sarfraz, V. Sharma, and R. Stiefelhagen, "Efficient parameter-free clustering using first neighbor relations," in *Proc. IEEE Conf. Computer Vision and Pattern Recognition*, 2019, pp. 8934–8943.

[5] B. McMahan, E. Moore, D. Ramage, S. Hampson, and B. A. y Arcas, "Communication-efficient learning of deep networks from decentralized data," in *Proc. Int'l Conf. Artificial Intelligence and Statistics*, 2017, pp. 1273–1282.

[6] Y. Tan, G. Long, L. Liu, T. Zhou, Q. Lu, J. Jiang, and C. Zhang, "Fedproto: Federated prototype learning across heterogeneous clients," in *Proc. AAAI Conf. Artificial Intelligence*, vol. 36, no. 8, 2022, pp. 8432–8440.

[7] J. Zhang, Y. Liu, Y. Hua, and J. Cao, "Fedtgp: Trainable global prototypes with adaptive-margin-enhanced contrastive learning for data and model heterogeneity in federated learning," in *Proc. AAAI Conf. Artificial Intelligence*, vol. 38, no. 15, 2024, pp. 16 768–16 776.

[8] X. Mu, Y. Shen, K. Cheng, X. Geng, J. Fu, T. Zhang, and Z. Zhang, "Fedproc: Prototypical contrastive federated learning on non-iid data," *Future Generation Computer Systems*, vol. 143, pp. 93–104, 2023.

[9] L. Wang, J. Bian, L. Zhang, C. Chen, and J. Xu, "Taming cross-domain representation variance in federated prototype learning with heterogeneous data domains," *Proc. Advances in Neural Information Processing Systems*, vol. 37, pp. 88 348–88 372, 2024.
